# Oil palm and gendered time use: A mixed-methods case study from West Kalimantan, Indonesia

**DOI:** 10.1016/j.forpol.2021.102682

**Published:** 2022-04

**Authors:** Dominic Rowland, Giacomo Zanello, Edy Waliyo, Amy Ickowitz

**Affiliations:** a Centre for Development Environment and Policy (CeDEP), SOAS, University of London, 10 Thornhaugh St, Bloomsbury, London WC1H 0XG, UK; b Centre for International Forestry Research (CIFOR), Jalan CIFOR, Bogor 16115, Indonesia; c School of Agriculture, Policy and Development, University of Reading, RG6 6EU, UK; d Kampus Gizi, Politeknik Kesehatan Kemenkes Pontianak (POLTEKKES), Jl. 28 Oktober, Siantan Hulu, Kota Pontianak, Kalimantan Barat 78242, Indonesia

**Keywords:** FFB, Fresh Fruit Bunch, KKPA, Kredit Koperasi Primer Anggota, NTFP, Non-Timber Forest Product, PIR, Perkebunan Inti Rakyat, WEP, Wild Edible Plants, Palm oil, Swidden, Indonesia, Time use, Gender, Forests

## Abstract

Measuring the social impact of oil palm requires the use of multiple metrics which capture different dimensions of well-being. To date, most studies have examined welfare outcomes at the household level, relying on a relatively narrow range of indicators. There is a need for a more diverse range of metrics to measure the social impacts of oil palm as well as more explicit accounting for study context and gendered effects. Here we demonstrate the utility of specialised time use methods used in combination with qualitative research to understand intra-household labour dynamics associated with oil palm adoption. We use a mixed-methods approach to investigate the role of smallholder oil palm plasma schemes on men and women's time use in Kapuas Hulu District, West Kalimantan. Time allocation is an important determinant of well-being as well as maternal and child nutrition and an indicator of women's empowerment and gender equality. We integrate the results from a fractional multinomial logistic regression of data from 603 individuals with qualitative findings on the subjective experience of time allocation, as well as, the causes, consequences and coping strategies to manage trade-offs in time allocation. We find that relative to non-oil-palm adopting swidden farmers, participation in oil palm plasma schemes is associated with more time spent in productive labour for both men and women, driven by off-farm labour on oil palm plantations. For women, increased time comes at the cost of reduced time spent in rest, leisure and sleep. Increased time spent in off-farm labour drives households to adapt agricultural production methods, changing cash crop production as well as accelerating swidden transitions. These changes alter gender dynamics and responsibilities within the household. Our results suggest that changes in time allocation may have significant consequences for women's well-being and gender equity. Women in the oil palm site experienced greater stress over time scarcity and employed coping strategies more frequently. Our findings indicate that time allocation could be used as an indicator of the effects of oil palm expansion and adoption on well-being and that potential effects of time scarcity on well-being, gender equity, and maternal and child nutrition should be considered by policy makers when making land use decisions.

## Highlights

• Measuring the social effects of oil palm expansion and adoption requires the use of multiple metrics.
• Participation in oil-palm plasma schemes among former swidden farmers was associated with increased time scarcity for women.
• Women in oil palm adopting households spent less time in rest and leisure activities and less time asleep.
• Time pressure drives land use change, accelerating swidden transitions and modifies household gender roles.

## 1 Introduction

Despite decades of research, there remains no consensus over the social and well-being effects of oil palm expansion on local communities in Indonesia (Meijaard and Sheil, 2019). Oil palm expansion and adoption has been associated with increased incomes, economic growth and poverty reduction (Edwards, 2019; Qaim et al., 2020). However, recent research suggests oil palm affects different dimensions of well-being in different ways, producing different outcomes depending on pre-oil palm environmental, economic and livelihood conditions. For example, the economic benefits of oil palm adoption appear to be most concentrated among communities who were previously market orientated, while formerly subsistence orientated communities may experience long-term negative effects (Santika et al., 2019a, Santika et al., 2019b). As well as baseline conditions, there is a need to more explicitly study the effects of different oil palm models. Smallholder oil palm is a broad categorisation, often imprecisely and erroneously assigned, which encompasses a wide range of partnership and contract farming models as well as independent and semi-independent farmers of varying scales (Jelsma et al., 2014; Schoneveld et al., 2019a; Nurhasan et al., 2020). As such, there have been calls for a more diverse range of metrics to measure the social impacts of oil palm as well as more explicit accounting for study context (Santika et al., 2019a, Ayompe et al., 2021, Meijaard and Sheil, 2019, Nurhasan et al., 2020, Reiss-Woolever et al., 2021, Santika et al., 2019b).

Only a small proportion of studies on oil palm in Indonesia have examined the effects of oil palm adoption on well-being from an explicitly gendered perspective (Li, 2015). Local case studies, however, suggest that oil palm adoption may have significant effects on a wide range of determinants of intra-household inequality and women's well-being (Julia and White, 2012; Li, 2015; Elmhirst et al., 2015, Elmhirst et al., 2017). Gendered effects of oil palm adoption include the restructuring of intra-household labour dynamics. Previous studies have shown how oil palm adoption changes labour participation and gender roles in on and off-farm activities (Bissonnette, 2013, Krishna et al., 2017, Kubitza et al., 2019, Maharani et al., 2019, Chrisendo et al., 2020). However, no previous study has attempted to quantify gendered dimensions of labour allocation across all aspects of daily life. To do so requires the deployment of specialised time-use methods able to capture the complexities of concurrent activities as well as to accurately record time spent in neglected categories of labour including domestic labour and caregiving, alongside rest and leisure activities.

Measuring time allocation is vital in understanding hidden dimensions of intra-household labour allocation – especially in providing evidence of the ‘invisible’ role of women's labour in agricultural livelihoods as well as the routinely underestimated burden of reproductive labour (domestic work and caregiving) (Doss, 2018). It also provides insights into a range of other social and well-being outcomes. Time use is increasingly used as a measure of women's well-being and empowerment (Alkire et al., 2013; Williams et al., 2016). Time allocation can also affect a multitude of health and nutrition outcomes of women and their dependants (Strazdins et al., 2011; Johnston et al., 2015; Williams et al., 2016).

This study is the first to explore time allocation in the context of Indonesian oil palm using validated time use research methods. Our quantitative analysis is coupled with results from qualitative research, collected over a period of seven months, which contextualises findings and offers an understanding of the potential pathways through which households reorientate labour towards oil palm based livelihoods. We aim to answer the following research questions:

1. How does the allocation of men and women's time differ between oil palm adopting and non-oil-palm adopting households?
2. What drives patterns of time allocation in different contexts?
3. What is the subjective experience of different patterns of time allocation?

## 2 Background and context

### 2.1 Oil palm and gendered labour allocation

Investigations into the gendered effects of oil palm have been limited by the lack of publicly available gender disaggregated data – with the majority of large-scale socio-economic analyses using household, village, or even district level data (Li, 2015). Focus on the household as the unit of analysis also stems from a long history of development narratives centred around households as the productive unit (Elmhirst, 2011; Li, 2015). While the majority of large-scale quantitative analyses have analysed oil palm at the household level, a number of case studies suggest that oil palm adoption may have adverse effects on intra-household inequality, land-ownership and women's well-being (Julia and White, 2012; Li, 2015; Elmhirst et al., 2015, Elmhirst et al., 2017).

One set of studies has focused on oil palm adoption among cash crop farmers in Sumatra, using econometric approaches applied to panel survey data (Kubitza and Gehrke, 2018; Kubitza et al., 2019; Chrisendo et al., 2020). Among rubber farmers in Sumatra who do not grow their own food, adopting oil palm as independent smallholders, reduced on-farm labour time for both men and women, but resulted in increased participation in off-farm labour For men only (Chrisendo et al., 2020). Men's increased participation in off-farm work is attributed to the relative labour-efficiency (labour-time per ha of land) of oil palm compared to rubber. The labour efficiency of oil palm frees up time for off-farm work (or oil palm expansion). Indeed, farm expansion and increased participation in off-farm work appear to be the main pathways by which oil palm adoption increases income among independent smallholders (Euler et al., 2016). No comparable effect was found for women, attributed to social and cultural norms restricting access to off-farm labour for women.

In contrast to studies among former market-orientated rubber farmers in Sumatra, studies of oil palm adoption among subsistence farmers in Kalimantan suggest that oil palm livelihoods may increase the overall burden of productive labour as households continue to manage diverse portfolio livelihoods (Julia and White, 2012; Bissonnette, 2013; Li, 2015; Elmhirst et al., 2016; Maharani et al., 2019; Toumbourou and Dressler, 2020). As subsistence farmers adopt oil palm, many subsistence farmers continue to self-produce the majority of their household's food – at least in the short term (Orth, 2007; Fortin, 2011; Dove, 2011; Li, 2015; Elmhirst et al., 2016, Elmhirst et al., 2017; Jelsma et al., 2017; Toumbourou and Dressler, 2020). Qualitative accounts in this context have shown a transition towards men becoming the primary source of household income, with women taking increasing responsibility for food production (Fortin, 2011; Julia and White, 2012; Li, 2015; Maharani et al., 2019; Toumbourou and Dressler, 2020).

### 2.2 Socio-economic, ecological, and political context

Situating research within the context of local agrarian and landscape transitions is vital to parse the contexts in which oil palm adoption and expansion may yield positive impacts from those where the impact may be negative (Tyson et al., 2018; Santika et al., 2019a, Santika et al., 2019b; Nurhasan et al., 2020). The term smallholder covers a wide variety of different oil palm models. However, both official statistics, and a sizable fraction of research studies, report smallholder oil palm as a single entity (Jelsma et al., 2017; Schoneveld et al., 2019a, Schoneveld et al., 2019b; Purwanto et al., 2020). Different oil palm models likely result in different social and economic outcomes, though lack of standardised methods, indicators and definitions has meant that few comparative research attempts have been made (Hidayat et al., 2015; Schoneveld et al., 2019a, Schoneveld et al., 2019b). Likewise, local livelihoods prior to oil palm adoption (such as market integration, degree of commercialisation) influence the impacts of oil palm adoption (Santika et al., 2019a, Santika et al., 2019b), as do regional economic, ecological and policy contexts (including local history of plantation agriculture and market infrastructure) as well as spill-over effects from nearby oil palm activity (Bissonnette and De Koninck, 2017).

#### 2.2.1 Smallholder models in Indonesia

Smallholder production accounts for 46% of area planted with oil palm and 37% of crude palm oil production in Indonesia (BPS, 2017). It is also the fastest growing sector of the Indonesian oil palm industry, estimated to reach 60% of plantation area by 2020 (Saragih, 2017). Smallholders lie on a continuum between farmers fully tied to corporate plantations (as participants in smallholder plasma schemes) to fully independent smallholders (IFC, 2013; Daemeter, 2015; Zen and Nibulan, 2018; Naylor et al., 2019). However, no distinction betwen types of smallholders are made in official statstics, making it hard to determine the extent of different models in different regions (Daemeter, 2015, BPS, 2017). Nevertheless, it is likely that independent smallholders are most prevalant in regions with long histories of oil palm development while regions with more recent oil palm expansion are likely to be dominated by participants in partnership schemes.

Independent smallholders are farmers who grow oil palm on their own land, selling fresh fruit bunches (FFBs). Most independent smallholders however, do not sell FFBs directly to mills, and informal ties and networks of middlemen mediate relationships between farmers and mills (Jelsma et al., 2017; Naylor et al., 2019). Independent smallholders consist of two types. They may be former participants in smallholder schemes who have paid off debt obligations to companies and now farm independently, or they may be farmers who independently adopted oil palm without assistance. In reality, many smallholders “*neither fit the legal nor popular definition of ‘smallholders’* ” (Jelsma et al., 2017). Legal ambiguity and lack of clear definitions can result in medium and large-scale farmers, city investors or proxy owners being classified as smallholder farmers. Only two studies have used primary survey data methods to estimate oil palm smallholder heterogeneity among independent smallholder farmers (Jelsma et al., 2017; Schoneveld et al., 2019a, Schoneveld et al., 2019b). While in different contexts, both studies highlight the degree to which independent smallholders are dominated by local elites and wealthy outside investors.

Participants in smallholder plasma schemes are tied to corporate plantations. Since their inception in the late 1970s, smallholder schemes have undergone several stages of evolution, with varying levels of state versus private sector control, financing mechanisms, and revenue sharing arrangements (IFC, 2013; Daemeter, 2015; Zen and Nibulan, 2018). All schemes, however, share certain common characteristics; they consist of a core company plantation “*inti*” surrounded by a collection of small parcels of smallholder oil palm “*plasma*” land. Plasma schemes can be categorised into two main groups. The oldest type of model, known as PIR (*Perkebunan Inti Rakyat*), are out-grower schemes where smallholders farm their own oil palm land (typically 2 ha). The scheme is intimately connected to transmigration schemes (although are also present in local communities). Financing and technical assistance is provided by the company, who buy FFBs from smallholders at set prices after deducting for loan repayments, input and other assistance.

Over time, out-grower models have become increasingly less generous, and since the 1990s they have been largely replaced by partnership models (Zen and Nibulan, 2018). In partnership models, smallholder land is pooled into a larger plantation managed by the company. In this model it may be hard to distinguish smallholder plantations from the main company estate. Participants receive a share of company profits, theoretically derived from the area of land that they have leased to the company. While there have been numerous iterations of this type of scheme the KKPA (*Kredit Koperasi Primer Anggota*) has become the dominant model since the 1990s, whereby the partnership arrangement is mediated through village cooperatives. Plasma plots – which in effect operate like shares in a company plantation – are frequently sold and resold, either to wealthier local smallholders, outside investors or back to the company (Cramb and McCarthy, 2016, Naylor et al., 2019, Semedi and Bakker, 2014). As such, many so-called independent smallholders may simultaneously own plasma plots, even if they themselves have never participated in plasma schemes (Schoneveld et al., 2019a, Schoneveld et al., 2019b).

#### 2.2.2 Smallholder oil palm in West Kalimantan

Different oil palm models are not evenly distributed with different types of models clustering in different islands, provinces and districts,^1^ reflecting the periods in which oil palm was first developed as well as local economic and geographical conditions. Precise data on the extent of different smallholder models in West Kalimantan is not available. Official data, which combines independent and plasma models, suggest that smallholder oil palm is increasing throughout Kalimantan. For example, 57% of planted oil palm area between 2005 and 2015 was classified as smallholder lands^2^ (Schoneveld et al., 2019a, Schoneveld et al., 2019b). However, smallholder oil palm is still relatively underdeveloped in Kalimantan relative to Sumatra (Purwanto et al., 2020). For example, in West Kalimantan 29.5% of the planted oil palm area is classified as smallholder oil palm, compared with 70.6% in Riau and 72.5% in Jambi (BPS, 2017). Different oil palm models are also unevenly geographically distributed throughout West Kalimantan, reflecting local histories of oil palm development, infrastructure development, transmigration and political and social opposition. In areas with more recent oil palm expansion, such as Kapuas Hulu Regency, oil palm expansion continues to be driven by the expansion of company oil palm, with less than 10% of oil palm area occupied by smallholders (Purwanto et al., 2020). The expansion of corporate plantations is accompanied by the growth of plasma schemes. (Hasudungan, 2018; Hasudungan and Neilson, 2020). Legally, since 2007, all corporate oil palm development have had to include local landowners in smallholder plasma schemes^3^ and many provinces and districts have similar laws (Potter, 2016, Jong, 2020a, Jong, 2020b, EIA, 2021). Within plasma schemes, there is a general trend away from out-grower models towards company managed models (Gillespie, 2016; Hasudungan, 2018). This is true not only for new plantations but also for existing plasma out-grower schemes as many companies have applied incentives and coercion to convert towards shareholder models (Dove, 2011; Potter, 2011; Gillespie, 2016; Hasudungan, 2018).

#### 2.2.3 Swidden – oil palm transitions in Kapuas Hulu

Oil palm expansion in Kapuas Hulu cannot be separated from broader agricultural transitions including the decline of swidden cultivation. Like similar swidden transitions across South-East Asia, swidden transitions in Kapuas Hulu are influenced by a combination of demographic, market and governance forces (Padoch et al., 2007; Fox et al., 2009). Swidden transitions often share certain characteristics such as the intensification of agriculture, a move from collective to individual land tenure, relocation of cultivation to less upland areas, changes in crops cultivated, market integration, official banning and/or controlling of traditional practices, and restricted land access (Dressler et al., 2016). Swidden transitions often result in shorter fallow times, replacement with permanent perennial crops and/or annual monocrops as well as the loss of customary land tenure (Mertz et al., 2009; Dressler et al., 2018).

Swidden transitions in Kalimantan pre-date the arrival of oil palm. Traditional Dayak economies are diverse and dynamic, shifting the allocation of resources (land and labour) year-by-year in response to household demands, market fluctuations and land availability (Dove, 1981, Dove, 1983; Colfer et al., 2015, Colfer et al., 2016). The dynamism of traditional swidden systems has allowed communities to respond to economic opportunities and Price fluctuations (Purwanto, 2018), economic shocks (Wadley and Mertz, 2005), population pressure (Padoch et al., 1998), as well as to political coercion and incentives (Wadley, 2007; Thaler and Anandi, 2017). Changes may also be temporary as communities adapt to the emergence (and subsequent declines) of economic opportunities such as logging (Wadley and Eilenberg, 2005; Heri et al., 2010; Purwanto, 2018), agarwood collection (Paoli et al., 2001) and artisanal mining (Shantiko et al., 2013).

Swidden transitions which pre-date oil palm differ from oil palm driven swidden transitions in the degree to which changes are irreversible. In Indonesia, oil palm planting cannot legally take place within a village boundary without the consent of local communities. The nature of village consent is complex, gendered and consists of asymmetrical power relationships.^4^ In consenting to oil palm development, communities, whether knowingly or not, give up claim to customary ownership of land on which swidden cultivation is centered (Clerc, 2012, Rietberg and Hospes, 2018). Abandoning swidden fields and traditional regeneration cycles, also reduces the ecological adaptations of swidden agriculture for the landscape, leading to reduced soil fertility, reduced ecosystem regulation of pests and increased dependence on chemical inputs (Labrière et al., 2015a, Labrière et al., 2015b; Imang et al., 2018). Swidden transitions may also be gradual, driven by households reorientating labour towards oil palm and finding traditional swidden production incompatible with the labour demands of oil palm livelihoods (Maharani et al., 2019).

## 3 Methods

### 3.1 Study approach

This study formed part of a wider investigation into the effects of oil palm on diets and nutrition funded by the Drivers of Food Choice Competitive Grants Programme (DFC) and led by the Center for International Forestry Research (CIFOR) (Purwestri et al., 2019; DFC, 2020). The study aimed to compare diets and nutrition of smallholder oil palm farmers (participants in smallholder plasma schemes) in villages with widespread adoption of oil palm with the livelihoods of predominantly subsistent swidden farmers in non-oil-palm adopting villages. We refer to these sites as Oil Palm (OP) sites and swidden sites respectively. Villages sampled in the study were selected carefully from an extensive list of possible villages based on preliminary qualitative research. We do not pretend that swidden villages exactly represent the pre-oil palm state in the oil palm villages. Rather, oil palm and swidden villages share historical similarities but have subsequently diverged in different ways. Thus, the swidden villages in this study represent a possible alternative trajectory to oil palm adoption.

Any cross-sectional study design based upon a comparison of oil palm and non-oil-palm adopting households raises the issue of potential endogeneity. It is possible that there may be inherent differences in villages which make them more or less likely to adopt oil palm which may also influence the allocation of time. The need for longitudinal studies to obviate this inherent weakness of cross-sectional designs and is discussed in section 4.6. However, in the absence of longitudinal data, we opted for this comparative approach (comparing households in oil palm adopting villages with households non-oil-palm adopting villages) as it reflects the way in which oil palm is adopted by plasma communities. For plasma agreements, consent is granted by village authorities on behalf of village residents and dividends, compensation and other forms of payments are collectively bargained (Andrianto et al., 2019; Yuliani et al., 2020). Additionally, this approach avoids the issue of survivorship bias inherent in other cross-sectional approaches – as households who abandon oil palm after adoption (and sell their oil palm land) are not excluded from the sample, while successful oil palm farmers (who may accumalte land by buying from unsuccessful farmers) are not over-represented in the sample. Further justification for this is approach, combined with potential sources of endogeneity are discussed in more detail in the supplementary data.

### 3.2 Selection of study villages

The selection of study villages was carried out based on extensive preliminary research using a combination of existing publicly available data, consultation with knowledgeable expertsand qualitative research. The objective was to find oil palm and non-oil-palm adopting villages that shared similar historical livelihoods at a baseline time prior to oil palm development in the region (i.e., before the 2000s). Several criteria were used for identifying candidate villages for inclusion in the study. All candidate villages were required at the baseline period to have (1) predominantly indigenous Dayak populations, (2) swidden agriculture and forest-based livelihoods; (3) comparable infrastructure and access to markets.

A list of potential villages were selected from all villages within Kapuas Hulu Regency using expert consultation and public data. Focus groups were then carried out in each of the potential villages focusing on historical (pre-year 2000) livelihoods, demographics, economic conditions. The final sample consisted of 13 oil palm and 13 non-oil-palm villages. In all selected villages, food production was produced via subsistence agriculture, primarily slash-and-burn rotational swidden rice cultivation. Livelihoods at the baseline period consisted of swidden agriculture combined with forest-based activities (hunting, fishing, collection of other NTFPs) as well as small-scale rubber agroforestry. No villages with extensive participation in logging or mining activities were included. Land tenure in all villages was historically based upon customary land ownership. In addition to these inclusion requirements, oil palm villages were required to have extensive community wide participation in oil palm plasma schemes. Only one publicly available dataset is available for the study villages at a time period prior to our historical baseline (BPS, 1996). While indicators are broad, this data supports our premise that the oil palm and swidden villages included in the study were broadly similar prior to the arrival of oil palm (Supplementary Data: Table A.6).

### 3.3 Respondent selection and data

As the primary study was focused on maternal and child nutrition, survey participants were households with children between the ages of 12-months and 5 years. In the swidden site, respondents were randomly selected from eligible candidates using a list of village residents meeting inclusion criteria provided by the local health service posts*.* In the OP site, the same selection process was applied, but with the additional criteria that respondent households were enrolled in an oil-palm plasma scheme. Our survey was a resurvey of a subset of the respondents, with an added survey of the husbands. Respondent women were selected randomly from the original survey list. Women's availability was high and there was unlikely to be any systematic bias in women's availability. In contrast, however, recruitment of men was more difficult. Men who worked extremely long hours were less likely to be available for surveys as were men who worked as temporary migrant labourers.

#### 3.3.1 Study timing and seasonality

Both qualitative and quantitative data was collected simultaneously over the course of seven months between January and August 2018. In Kapuas Hulu, swidden cycles generally consist of harvesting between February and March, land clearing and planting around July and August and planting around August and September (Supplementary Data: Table A.1). Due to resource constraints, it was not possible to constrain the survey to a single season and obtain a sufficiently large sample. To reduce the risk of seasonal bias, surveys were conducted alternately in oil palm and forest villages. No surveys were carried out with households during labour peaks in the swidden cycle (harvests, planting, burning seasons) or during religious or cultural festivities. However, a control was included in the analysis if other villagers were engaged in these activities. The lack of seasonal data is a major limitation of this study (discussed in section 4.6). However, the data captured represents a reasonable approximation of household activities outside of periods of peak labour demand.

### 3.4 Quantitative data collection

A time use questionnaire was developed consisting of both a quantitative time use recall survey and related time use questions. The time use survey module was added to an existing socio-economic and livelihoods survey administered to the male household head and an existing questionnaire focused on diets and food environments given to their spouse. In total, the time use module was carried out with a sample of 295 men and 336 women. Formative research in the sites indicated that only Sundays were taken off from formal work, and that routines were similar on the other six days. As the survey focused on activities in the preceding 24-hours, surveys were not administered on Mondays.

We adapted a 24-hour recall time use survey validated for a wide range of rural agricultural contexts as part of the Women's Empowerment in Agriculture Index (WEAI) (Alkire et al., 2013) by adding locally relevant activities such as hunting, fishing, and collecting NTFPs as well as a free text option for ‘other’ activities. The survey records up to two concurrent activities for each 15-minute block of time in the preceding day, with enumerators classifying activities as primary or secondary. Primary activities are the activities which were the objective of the time-block while secondary activities are those which were done concurrently with the primary activity. The inclusion of secondary activities is important as significant burdens of labour – especially reproductive labour such as childcare – can be missed when this is not considered. The time use survey was administered separately for men and women as part of a wider questionnaire by trained local enumerators. To reduce recall bias, enumerators used the Day Reconstruction Method (Kahneman et al., 2004), to first outline the major events in the respondents' previous day.

Following enumerator training, a pre-pilot test was conducted using cognitive interviews to improve question phrasing and technique. In addition, we validated the time-use recall survey with a small sample of participant “follows” with women in the swidden site to check recall accuracy and recall bias. Enumerators accompanied women from early morning until before women went to bed, with a different enumerator conducting the recall survey the following day. Despite a small sample size, the validation exercise indicated good overall recall accuracy using relatively broad activity categories, and no systematic under or over reporting of any category.

### 3.5 Qualitative data collection

Focus group discussions covered a wide range of topics including agricultural practices, forest use, land use and land use change, time allocation and household decision making. The time allocation and household decision making component focused on the subjective experience of time allocation and household approaches to managing time and making time-related decisions. Women-only focus groups were carried out in each of the 26 study villages. Focus groups consisted of between 10 and 12 women and covered a broad spectrum of different ages and primary livelihood activities. Focus groups with women had an emphasis on time allocation and labour including many aspects of reproductive labour such as food acquisition, cooking, childcare and other domestic activities. Mixed-gender and male-only focus groups were carried out in 10 villages, split evenly between the forest and OP sites, focusing on recent histories of economic, agrarian and land use change.

In addition to focus groups, we conducted key-informant interviews with 42 men and 29 women using semi-structured interview guides. Key informant interviews with women were split between general interviews focusing on women's livelihoods and time allocation and more detailed interviews focusing on reproductive labour.

### 3.6 Analysis

#### 3.6.1 Quantitative analysis

Rather than modelling absolute times spent in different activities, we elected to model the share of times allocated to activities. This approach is preferable to modelling absolute times as it reflects the inherent trade-offs between activities. We tailored a Fractional Multinomial Logit (FML) model developed by Mullahy (2015) and used by Picchioni et al. (2020) in the context of time-use studies with co-variates relevant to the local context. The methodology allows for the calculation of marginal effects that can be interpreted as trade-offs between time allocated in activties, keeping the daily allocation of time constrained to 24-hours in a day. The econometric specification is:

(1) $$\left[\begin{array}{c} y_{t}^{o}=\beta_{0}+\beta_{1}\mathrm{OIL}\_\mathrm{PALM}\times \mathrm{SEX}+\beta_{2}\mathrm{IN}D+\beta_{3}\mathrm{HH}+\beta_{4}\mathrm{CONTROLS}+\varepsilon \;\\ y_{t}^{a}=\beta_{0}+\beta_{1}\mathrm{OIL}\_\mathrm{PALM}\times \mathrm{SEX}+\beta_{2}\mathrm{IND}+\beta_{3}\mathrm{HH}+\beta_{4}\mathrm{CONTROLS}+\varepsilon \;\\ y_{t}^{r}=\beta_{0}+\beta_{1}\mathrm{OIL}\_\mathrm{PALM}\times \mathrm{SEX}+\beta_{2}\mathrm{IND}+\beta_{3}\mathrm{HH}+\beta_{4}\mathrm{CONTROLS}+\varepsilon \\ y_{t}^{p}=\beta_{0}+\beta_{1}\mathrm{OIL}\_\mathrm{PALM}\times \mathrm{SEX}+\beta_{2}\mathrm{IND}+\beta_{3}\mathrm{HH}+\beta_{4}\mathrm{CONTROLS}+\varepsilon \;\\ y_{t}^{s}=\beta_{0}+\beta_{1}\mathrm{OIL}\_\mathrm{PALM}\times \mathrm{SEX}+\beta_{2}\mathrm{IND}+\beta_{3}\mathrm{HH}+\beta_{4}\mathrm{CONTROLS}+\varepsilon \; \end{array}\right)$$

Where *y*_*t*_ represents the ratios of time allocated in a day in off-farm activities, agriculture and forest work, reproductive labour, leisure activities and sleep and rest ($y_{t}^{o}=\frac{t_{o}}{1440},\;y_{t}^{a}=\frac{t_{a}}{1440},\;y_{t}^{r}=\frac{t_{r}}{1440},\;y_{t}^{p}=\frac{t_{p}}{1440}$ and $y_{t}^{s}=\frac{t_{s}}{1440}\;$respectively, being the sum of time spent in the different activities (*t*_*o*_, *t*_*a*_, *t*_*r*_, *t*_*p*__*,*_*t_s_*) equal to 1440 min (24 h)). As our primary outcome of interest is the share of time spent in different activities, we weighted secondary activities and primary activities. In time blocks with both a primary and secondary activity, the primary activity was allocated 80% of the time (12 min) and the secondary activity was allocated 20% of the time (3 min). A sensitivity analysis was conducted to compare different proportions (Supplementary Data).

Off-farm activities (*t*_*o*_) are defined as all income generating activities which do not take place on the respondent’s household farm, for which they receive financial renumeration. While this category consists primarily of waged agricultural labour (both oil palm and non-oil-palm), it also includes non-agricultural salaried positions (e.g., teachers, civil servants, office jobs) as well as independent and household business activities (e.g. shops or handicrafts). Agricultural and forest-based activities (*t*_*a*_) is defined as time spent in labour relating to a household's farm production – whether for self-consumption or for sale – and includes both swidden agriculture and cash crops such as rubber or pepper. This category also includes all collection of forest products such as wild edible foods both for own-consumption and sale. Reproductive labour (*t*_*r*_) includes domestic labour in the home (cooking, cleaning, household chores) and outside the home (food shopping) as well as caregiving activities. Leisure and personal time (*t*_*p*_) is time engaged in recreational and leisure activities as well as time taken for personal care (e.g., washing, personal hygiene). Finally, sleep and rest time (t_s_) includes both sleep at night as well as during the day. Based upon standard methodology from the International Classification of Activities for Time-Use Statistics (ICATUS) (UNSD, 2016) we include time travelling to an activity within time allocated to that activity.

Our focus in the estimation of (1) is on the vector ***OIL***_***PALM × SEX*** that includes the full factorial interactions between sex (male and female) and a dummy variable capturing whether the village is in a forest or oil palm plasma site. The vector ***IND*** includes individual characteristics (age and education), while the household characteristics are captured in the vector ***HH***. These include household composition, wealth, and whether the spouse had waged work. Finally, control variables (vector ***CONTROL***) include farm characteristics (land size, farm diversity, use of inputs). While no surveys were conducted while households were engaged in peak-labour demand swidden activities (planting, harvesting, slash and burning), a dummy variable was included if the survey was conducted during a month when such activities are common in the swidden cycle.

The model was estimated in Stata with command FMLOGIT (Buis, 2008). The estimations control for autocorrelation among the outcome variables, heteroskedasticity, and non-linearities. Standard errors throughout are clustered at household level.

#### 3.6.2 Qualitative analysis

Transcripts of interviews and focus group discussions were translated from Bahasa Indonesia to English and a subset were back translated to Bahasa Indonesia to validate the translation process. Thematic analysis of qualitative data was carried out in NVivo 12 using a combination of deductive and inductive approaches (Braun and Clarke, 2006). Development of themes followed a multi-phase process as described by Nowell et al. (2017) beginning with codes generated from broad a priori themes relating to time allocation, intra-household allocation of time, time-saving strategies and technologies alongside with new codes added inductively from the data. The final codebook and descriptions of themes is provided in Table A.5 (Supplementary Data).

## 4 Results

### 4.1 Quantitative results

#### 4.1.1 Household and individual characteristics

The final data set consisted of 603 Individuals (200 household pairs where data was available for both men and women from the same household, as well as 65 individual men and 138 individual women). Individuals in the swidden site made up 45.1% of the sample with the remaining 54.9% coming from the OP site. In the swidden site, participants were predominantly female (57.7%) while in the OP site, males and females were equally represented. The descriptive statistics of the households are reported in Table 1. While we do not observe differences in the land endowments, households in the OP sites are wealthier and tend to be smaller, with fewer younger and older members. Table 2 shows the individual characteristics of the respondents. On average, women in the OP site were younger than women in the swidden site and significantly better educated.

**Table 1:** Household characteristics, by location.

	Swidden (*n* = 272)		Oil palm (*n* = 331)		
	Mean	SD	Mean	SD	Difference
*Demographic*					
Female particpant (y/n)	0.58	0.49	0.50	0.50	0.08*
No. children <14 yrs	2.51	1.12	2.27	1.14	0.24**
No. children 14-18 yrs	1.52	1.15	1.42	1.02	0.10
No. adults >60 yrs	0.28	0.57	0.16	0.44	0.12***
*Socio-Economic*					
Female employment	0.44	0.50	0.89	0.32	−0.45***
Land area (ha)	1.66	1.93	1.29	4.17	0.38
Wealth (asset index)	−0.73	1.27	0.82	1.68	−1.55***
*Farming*					
Hired labour (y/n)	0.75	0.43	0.65	0.48	0.10**
No rice (y/n)	0.03	0.17	0.18	0.39	−0.15***
Fertilizer on rice field (y/n)	0.00	0.07	0.20	0.41	−0.19***
Pesticide on rice field (y/n)	0.13	0.34	0.24	0.43	−0.11***
Herbicide on rice field (y/n)	0.12	0.33	0.13	0.34	−0.01
Chemical input on rice field (y/n)	0.36	0.48	0.29	0.45	0.07
Grows rubber (y/n)	0.53	0.50	0.21	0.41	0.31***
Grows pepper (y/n)	0.04	0.19	0.21	0.40	−0.17***

Notes: *** 0.1% significant, ** 1% significant, * 5% significant.

**Table 2:** Individual characteristics, by sex and location.

	Men (*n* = 281)					Women (*n* = 322)				
	Swidden (*n* = 115)		Oil palm (*n* = 166)			Swidden (*n* = 157)		Oil palm (*n* = 165)		
	Mean	SD	Mean	SD	Difference	Mean	SD	Mean	SD	Difference
Age (years)	31.01	4.68	29.93	5.81	1.07	29.32	5.12	27.72	5.57	1.60*
Primary education (%)	0.49	0.50	0.67	0.47	−0.18*	0.48	0.50	0.76	0.43	−0.29***
Middle school education (%)	0.30	0.46	0.19	0.39	0.11	0.19	0.39	0.10	0.30	0.09*
High school education (%)	0.22	0.41	0.14	0.35	0.07	0.33	0.47	0.14	0.35	0.19***
High labour season (%)	0.65	0.48	0.67	0.47	−0.02	0.49	0.50	0.61	0.49	−0.12

Notes: *** 0.1% significant, ** 1% significant, * 5% significant.

#### 4.1.2 Time use

Mean shares of time for each group are reported in Table 3. Shares of time are the proportion of a respondent's time engaged in a particular activity over a day (24 h, 1440 min) and can range from 0 (no time spent in activity) to 1 (all the respondent's time spent in activity)^5^. On average, men spent most of their awake time in productive activities. While in forest areas the work was predominantly on their own farm or the collection of forest products; in oil palm areas, wage work took on average 30% of their time (equal to more than seven hours a day). Men's time in reproductive work, leisure and sleeping was similar in the two sites. This contrasts with the pattern of time use of women. On average, women in the oil palm sector worked two hours more than those in the swidden sites and spent 72 min less on leisure and personal activities and had 45 min less sleep.

**Table 3:** Allocation of time use, by sex and location. Results are reported as mean shares of time.

	Men					Women				
	Swidden		Oil palm			Swidden		Oil Palm		
	Mean	SD	Mean	SD	Difference	Mean	SD	Mean	SD	Difference
Wage Work	0.12	0.15	0.30	0.10	−0.18***	0.06	0.10	0.25	0.11	−0.19***
Agriculture and Forest	0.20	0.14	0.05	0.07	0.15***	0.13	0.12	0.03	0.06	0.10***
Reproductive Labour	0.07	0.08	0.07	0.06	0.01	0.19	0.09	0.18	0.10	0.02
Personal and Leisure	0.26	0.08	0.25	0.07	0.01	0.26	0.08	0.21	0.05	0.05***
Sleep	0.34	0.04	0.34	0.04	0.01	0.35	0.03	0.33	0.03	0.02***

Notes: *** 0.1% significant, ** 1% significant, * 5% significant.

#### 4.1.3 Regression results

**Fig. 1:**
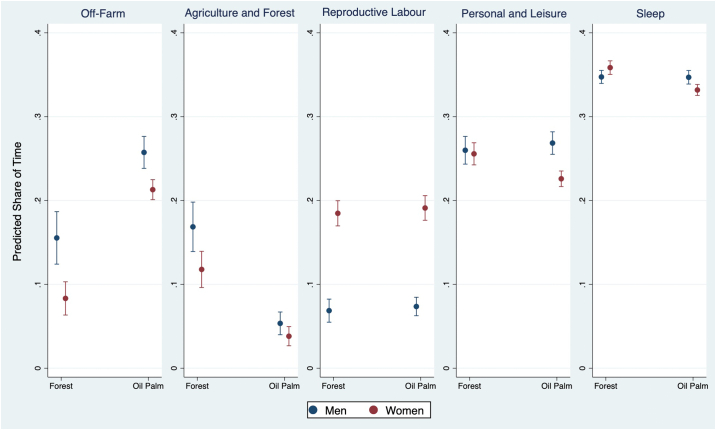
Predicted shares of time spent in different activities for forest and oil palm areas, by sex. Notes: The bar plots show mean and 95% confidence intervals of time shares, predicted from the fractional multinomial logit model.

Regression marginal effects are reported in Fig. 1. Marginal effects of covariates on time shares in activities are reported in Table A.2 (Supplementary Data). The significance level of the trade-offs by sex (within) and areas (between) of predicted shares of time spent in different activities are reported in Table 4. Both men and women in the OP site spent less time in agricultural and forest activities and significantly more time in off-farm labour compared with the swidden site. However, the reduction in the share of time spent in agricultural and forest-based activities was greater for men than for women. Women spent 12% more time than men in reproductive work, without any significant difference between sites.

**Table 4:** Statistical difference of predicted shares of time spent in different activities, by sex (within) and areas (between).

	Wage work	Agriculture and forest	Reproductive labour	Personal and leisure	Sleep
Male (baseline) vs Female					
Swidden	−0.07***	−0.05***	0.12***	−0.00	0.01*
Oil Palm	−0.04***	−0.02*	0.12***	−0.04***	−0.02***
Swidden (baseline) vs Oil Palm					
Male	0.10***	−0.12***	−0.00	0.01	−0.00
Female	0.13***	−0.08***	0.01	−0.03**	−0.03***

Notes: *** 0.1% significant, ** 1% significant, * 5% significant.

The different pattern of activities between each site reflects the substitution of activities in the oil palm and swidden site. In non-oil-palm areas, the additional time women engage in reproductive work is compensated by less time in off-farm work compared to men. However, for women in oil palm areas, less time is available for leisure and personal activities and sleep compared to men. Compared with the swidden site, both men and women allocate more of their time to wage work and less of their time to agricultural production. However, for women, the additional time that spent in waged work in oil palm areas is associated with a reduction of personal and leisure time.

### 4.2 Qualitative results

Focus groups and key informant interviews revealed clear differences in the experience of time allocation, time pressure, trade-offs and coping strategies. Table 5 summarizes the key differences between sites, while themes are illustrated by additional quotes in Table A.4 (Supplementary Data). Major themes were classified into broad groups including the subjective experience of time allocation, causes of trade-offs in time allocation, managing trade-offs in time allocation, labour-saving efficiencies, coping strategies for time pressure and efficiency and consequences of patterns of time allocation.

**Table 5:** Emergent themes and descriptions.

Theme	Oil palm	Swidden
**Subjective Experience of Time Allocation**		
Periodicity of time pressure	• Time pressure is consistent without periods of rest.	• Cyclical periods of intensity followed by rest periods.
Physical and mental exhaustion	• Men experience mainly physical exhaustion. • Women experience both physical and mental exhaustion. • Women report stress at managing competing demands of caring for husband and children alongside own-production and waged labour.	• Time pressure is experienced as busyness not exhaustion.
Social dimension of time allocation	• Women perceive few opportunities to socialise with other women outside of festivities and holidays. • Men and women do not see each other for much of the day. • Women spend farming time alone or while carrying children. • Socialising for men occurs at sides of roads during breaks and following waged labour as well as evenings. • Women's evenings are primarily filled with domestic activities such as preparing the next day's breakfast. Women carry out domestic duties instead of socialising during plantation breaks.	• Men and women work together all day as household unit. • Socialising occurs in later afternoons and evenings. • Social time may be constrained for women in evenings due to cooking and cleaning duties.

**Causes of trade-offs in Time Allocation**		
Flexibility of income generating activities	• Fixed contracted hours daily. • No flexibility to account for seasonal demands of swidden. • Time of day required for oil palm labour conflicts with rubber production.	• Work outside village (e.g., Agarwood seeking, temporary oil palm labour) can be arranged around low labour demand periods of swidden agriculture. • Time spent in rubber can be increased, decreased, or paused according to seasonal demands of swidden and need for household income. • Time spent in rubber is not limited by area of land planted by rubber due to profit sharing arrangements.

**Managing Trade-Offs in Time Allocation**		
Household Decision Making	• Aim to maximise men's time spent in income producing activities as men are paid more. • Women not able to work as long due to childcare and domestic duties. • Men aim to take on well-paid over-time work.	• Household decision making based upon short-term need. • Income production can be upscaled on demand through spending or less more time in rubber to adapt to own production activities.
Dual Livelihoods	• Income from oil palm is insufficient to allow households to purchase an entire family's food supply.	• Dual livelihoods mitigate risks through diversification.

**Labour-Saving Efficiencies**		
Labour saving modifications to rice production	• Relocation of fields closer to villages and roads reduces travel time. • Reduced fallow length and reusing plots in successive years reduces extent of cutting and burning trees. • Use of tree poisons reduced need for cutting and burning trees. • Increased use of fertilizers compensates for reduction in soil fertility from relocating and reducing swidden cycles. • Use of pesticides and herbicides reduces pests and weeds and compensates for loss of natural swidden adaptations. • Observed tendency for dependency on chemical inputs to increase over time.	• Selection of swidden site is the most important factor in determining productivity and labour demands of rice production. • Use of chemicals varies by villages but when used is used sparingly. • Those most likely to use chemicals on rice production are those who already purchase chemicals for cash crops such as chillies as well as some rubber farmers. • Application of chemicals is seen as poor value for money when same effects can be achieved through swidden rotation. Money is seen as best spent on goods and services which cannot be grown. • Some villages have experimented with but then abandoned more intensive agriculture.
Crop choice	• Rubber not grown due to time constraints. • Pepper grown instead of rubber which is more capital intensive but requires less time.	• Many crops are perennials which can be harvested as and when needed or available. • Wild and semi-cultivated foods harvested from forests and fallows.

**Coping Strategies**		
Activity bundling childcare with other activities	• Ability to carry out childcare whilst carrying out other tasks is constrained by children not being allowed on oil palm plantations. • Plantations, villages and surroundings are not considered safe for children to play unsupervised or casually supervised.	• Older children can play casually supervised while carrying out agricultural work or else play unsupervised around the village. • Younger children can be carried on slings during agricultural work.
Outsourcing childcare	• Grandparents are preferred source of childcare while women work in plantations. • If grandparents are not available, mothers can leave children with company employees in informal day-care arrangements or with security guards. • Payment for day-care is deducted from women's wages and women leave food with which to feed children. • New mothers stay at home only if household is wealthy enough.	• New mothers stay at home where possible. • Children are kept with mother and family members at all times. • Childcare takes place alongside other tasks.
Reducing time acquiring and cooking food	• Purchasing foods from mobile vendors. • Consumption of pre-prepared foods from vendors (occasional). • Collection of wild foods. Ferns collected while walking to and from plantations. Wild ferns and sweet potato collected from edges of plantations. • Selection of quicker foods to cook (vegetables, instant noodles, eggs) and reducing consumption of foods which take longer (meat).	• Opportunistic collection of wild foods while collecting rubber, farming or walking to and from fields. • Food acquisition as a leisure activity e.g., hunting / fishing. • Selection of quicker foods to cook (vegetables, instant noodles, eggs).

**Consequences**		
Gendered effects of swidden transition	• Reduced need for male labour in cutting and burning season as fewer large trees to clear. • Reduced need for male labour in carrying heavy loads and harvests due to shorter distances and access via motorbike. • Women able to take on some clearing work with chemical inputs. • Men continue to mix chemical inputs due to better ‘knowledge;’.	• Men and women work side-by-side but in different tasks. • Women leave fields earlier than men to return home to begin cooking.
Traditional land tenure and labour arrangements	• Transition away from customary ownership of land and emergence of land market. • Reduced use of traditional reciprocal labour exchange agreements.	• Traditional reciprocal labour-exchange arrangements are still widely used. Labour is exchanged in wide family units or more official farmers groups. • Customary land tenure governed by customary inheritance rules. • Former fallows closer to villages converted to rubber follow less traditional customary tenure arrangements.
Gender roles in food production	• Women primarily responsible for day-to-day food acquisition and cooking. • Day-to-day agricultural activities are vegetable gardening (women) and livestock management (men and women). • Men and women equally responsible for rice production but with different roles. • Men's tasks are more physical in nature or require technical knowledge of chemicals and machinery.	• Women responsible for cooking but food acquisition joint enterprise. • Food acquisition is gendered. Men hunt, fish and collect heavy WEPs which require carrying (e.g., palm hearts). Women engage in opportunistic collection of WEPs and harvest vegetables from agriculture. • Men and women equally responsible for rice production but with different roles. • Men's roles are more physically demanding such as cutting and clearing land and carrying heavy loads.

#### 4.2.1 Subjective experience of time

*“The [breaks] are not enough, because I come home from work at 2. There is a bit of rest, sometimes, but 3 o'clock, it must be food preparation, looking for clean water to drink.”* (OP_KI_F_V10)

*“In the evening working too, working the evening. If you rest when you are tired, it is impossible. We are pushed for time. If you are resting the work is not continuing.”* (OP_KI_F_V9)

Both men and women reported more severe time pressure in the OP site, compared with the swidden site, though the experience of time pressure differed with men reporting physical exhaustion from oil palm labour but women reporting stress and tiredness due to managing competing demands on their time. The periodicity of time pressure differed between sites. Unlike the OP site, where time pressure was seen as persistent, time pressure in the swidden site was cyclical, with periods of intense labour almost always followed by periods of low labour combined with rest and recuperation.

*“Even on Sundays we sometimes go to the fields. There is no rest…”* (OP_KI_F_V9)

A key emergent theme was the importance of breaks and socialising. Women in the OP site took only one short rest period between returning from the plantation and going out to household fields. During official breaks in plantation labour, women returned home (often against the instructions of supervisors) to begin cooking and carrying out other domestic duties. Women also forwent leisure time in the evenings to begin preparing breakfast so that men could wake up and immediately eat before going to work. The contrast with the swidden site is clear. In the latter, women and men spend much of the day together and both take breaks between activities and periodic rests during work.

*“By the evening we are already tired. We are already sleepy. We do not have energy [to socialise] and need to wake up in the morning”* (OP_KI_F_V12)

#### 4.2.2 Trade-offs in time allocation and coping strategies

Respondents in focus groups and key informant interviews were asked to free list trade-offs in time and labour allocation. Trade-offs were defined as conflicts between activities such that more time spent on one activity meant less time spent on another. Trade-offs identified were collated and categorised into broad themes. The most common categories of trade-off identified by both men and women in both sites concerned time spent in income production and food production. Women in both sites also identified several trade-offs between time spent in productive labour and reproductive duties.  

*Income producing and food producing activities:*

*“Finding land is not yet a difficulty. For us, the only difficulty is finding time”* (OP_KI_F_5)

Respondents in both the swidden site and the OP site identified trade-offs in time allocation between food-producing and income generating activities. An emergent theme from discussions was the degree of flexibility or inflexibility of income generating activities. In the swidden site, time spent in income generating activities could be increased or decreased in response to short-term household needs and seasonal swidden cycles. While access to off-farm waged labour was limited, income could always be obtained by allocating more time to rubber collection or the collection and sale of NTFPs.^6^ Likewise, both these activities could be reduced or paused without consequences such as reduced yields or losing a job.^7^ In contrast, the primary source of income in the OP site, waged plantation labour, required fixed shifts of 4–7 h per day, six days a week, at a set time of the day. Rubber cultivation was seen as incompatible with oil palm labour both for the time it required to collect and due to conflicting schedules.^8^ As a result, households switched cash crop production from rubber to pepper. Other income generating activities such as hunting or collecting NTFPs were also considered too time consuming to be compatible with oil palm labour.

*“It [fields] used to be far because we walked. Now it feels close for us because we use a motorbike. Now people think they don't want to have a field far away because it takes time. Now people think, because they are busy working, they will farm closer to their homes so they are easy to maintain, easy to monitor”* (OP_KI_FGD_V5)”

Respondents in the OP site also cited conflicts between traditional swidden agriculture and plantation labour. Most respondents felt unable to abandon food crop production and rely solely on income from oil palm labour. However, swidden was considered too time consuming – primarily because of the time necessary to travel to and from fields located far from the village – but also due to seasonal labour requirements around harvest, land-clearing, and burning seasons. Management of this conflict was achieved through reducing time spent in food crop production by making changes to rice production. By relocating fields away from steep slopes to more accessible locations close to villages or roads, households were able to reduce time spent walking to and from rice fields. This also meant that motorbikes could be used to access fields quickly, as well as transport heavy goods. In some villages, swidden cycles had been lost entirely, with households switching to permanent rice fields in naturally occurring hollows which flooded during the rainy season. This transition reduced the labour required annually to prepare fallows for planting by cutting, clearing and burning. Not all households had abandoned swidden cycles altogether; many households simply reduced the frequency of field rotation and reduced the length of fallow periods.

*“For example, if we do it manually, traditionally, it takes one month. But now we use herbicides, with that it is much faster, for example, 2 weeks becomes two days”* (OP_KI_M_V9)

The move away from traditional swidden systems was made possible using income from oil palm labour used to purchase chemical inputs, as well as increased access, knowledge and experience using chemicals acquired from plantation labour. Respondents frequently cited declining soil fertility and increased pests after abandoning fallow systems. Use of chemicals allowed households to overcome these barriers. Chemical inputs also, in combination with reduced fallow length, reduced the need for certain types of labour including cutting, burning and clearing land (due to younger forest regrowth), thus reducing labour required during peak swidden seasons. Income from plantation labour also enabled labour to be hired during peak periods. Using outside labour was also common in the swidden site – but typically took the form of reciprocal labour exchanges between households and kin (g*otong royong)*. This practice, though common before, had died out in the OP site as it required taking off workdays in plantation labour. For daily labourers, contractual terms allowed both men and women to take unpaid time off as required. However, women were more likely than men to take this time off during peak rice labour seasons. For men, the option of hiring outside labour was seen as preferable to taking time off if daily plantation wages in were greater than the cost of hiring labour.  

*Productive, reproductive and leisure time:.*

*“Yes sometimes, if we are busy. We will skip the rest”* (OP_KI_F_V8)

Women in both sites reported challenges in managing the competing demands of reproductive labour such as cooking and childcare alongside productive labour in on-farm and off-farm work. Women in both sites reported sacrificing leisure time and sleep to meet the demands of domestic labour and caregiving. Women had similar strategies in both sites for coping with time pressure and time scarcity – but women in the OP site reported using these strategies more frequently. For example, one strategy was using evenings to cook and prepare meals for the next day. While this was seen as an occasional necessity in the swidden site, it was a daily practice for many women in the OP site.

*“We wake up earlier [than husbands], around 4 we wake up, we prepare breakfast and so on for our husbands… so they will be able to directly eat breakfast and immediately go to work”* (OP_KI_F_V4)

“*You don't have time because … when we come home from work, we work again to take care of our husbands”* (OP_KI_F_V7)

Other common strategies to cope with time scarcity were reducing the time spent acquiring and cooking food by purchasing food (mainly OP site), collecting wild foods close to a respondent's activity space (both sites), selecting quicker foods to cook (both sites) or through using faster cooking fuels (OP site only). Finally, out-sourcing of childcare to other family members such as grandparents was common – but considerably more frequent in the OP site. Some childcare was also outsourced to oil palm company employees when other family members were unavailable in formal or informal company supplied (but fee-paying) childcare.

#### 4.2.3 Gender roles and allocation of time and labour

*“Men can work in all kinds of jobs for the oil palm company because men are more able and men only work for companies. They do not need to do other work, such as taking care of household activities, farming, etc.”* (OP_KI_F_V7)

Men had access to a wider range of jobs in the oil palm sector, including the best paying jobs. As daily labourers, men were paid more per hour and worked longer hours than women. Both men and women respondents cited greater knowledge and capacity with machinery as well as more physical tasks as the reason for the pay differential. The pay differential was commonly cited as a reason why men preferred to work long hours. Men also had access to overtime work that women did not (such as truck drivers and security guards). It was common for men to combine plantation labour with some over-time work leaving little time for other tasks during the day. As a result, women carried out most of the farming during the six-day working week.

*“My husband leaves early in the morning and comes back home at night, or late afternoon. Sometimes when he works as a driver he has to work late at night. So he doesn't have time to work with me unless there is a day off. It is like this, when men are busy with their work activities, automatically the women do the farming.”* (OP_KI_F_V6)

While most women did not have the option to work longer hours, it was also not seen as desirable. Both men and women, cited caregiving and domestic duties as a reason why longer women could not work longer hours. In both sites, women were the primary care givers and took on the majority of reproductive labour including cooking and domestic work. Compared with the swidden site, women in the OP site took on a wider range of roles in food producing agriculture compared with women in the swidden site (Table 6).

**Table 6:** Consensus views on gender roles from focus group discussions. X = job normally done by gender, − = not present in site, (x) = occasional but not common.

		Swidden		Oil Palm	
		Men	Women	Men	Women
Plantation	Operating machinery	–	–	X	
	Applying chemicals	–	–		X
	Mixing chemicals	–	–	X	
	Harvesting FFB from palms	–	–	X	
	Picking fruits from floors	–	–		X
	Loading trucks and wheelbarrows	–	–	X	

Other off-farm	Company office jobs	–	–	X	
	Oil palm mill work	–	–	x	
	Supervisor positions	–	–	X	
	Village Officials	X	X	X	(x)
	Teachers, nurses, midwives etc.		X		X
	Truck drivers	–	–	X	

Business	Local shop	X	X	X	X
	Trading and transportation	X		X	
	Skilled Trades	X		X	
	Handicrafts		X		X

Own production	Planting	X	X	X	X
	Weeding		X		X
	Harvesting (Rice)	X	X	X	X
	Harvesting (Vegetables)	X	X		X
	Applying Chemicals	–	–	(x)	x
	Building huts and shelters	X		–	–
	Clearing Land	X		X	X
	Carrying and transporting	X		X	X

Cash crop	Rubber tapping	X	X	–	–
	Pepper planting and harvesting	–	–	X	X
	Commercial vegetable gardens	X	X	–	–
	Cash crop weeding and maintenance	X	X		X
	Cash crop harvesting	X	X	X	X

Forest	Hunting	X		X	
	Fishing	X		X	
	Collecting Wild Fruits and Vegetables		X		X
	Sale of NTFPs	X		–	–

Women were ultimately responsible for day-to-day food acquisition in both sites but men were more heavily engaged in the swidden site. Growing vegetables was only carried out by women in the OP site and was the most frequent reason to visit fields. Growing rice remained a joint household responsibility, although men did not dedicate much time to this task during workdays. Most of men's labour in rice fields was carried out on Sundays and holidays. Most income was jointly produced by swidden household, The exceptions were Agarwood collection and temporary migratory labour carried out by men, and handicrafts produced and sold by women. Rubber was sold to local traders jointly by the household, often against credit for food. NTFPs (excluding Agarwood) were also sold by both men and women regardless of who collected them. In contrast, women's income in the OP site, though important for the household economy, was considered supplementary to men's.

*“Both [men and women] try to earn money. Only it is more for the men, the men have to earn money, but she only helps. If she can get money, it is okay. But if she can't get money then she will think - he must go earn money”* (OP_KI_F_V3).

A noticeable contrast between sites was the degree to which households operated as a unit with members working alongside each other. In the swidden site (and prior to adopting oil palm in the OP site), men and women spent most of the working day time working side-by-side – first in rubber fields and then in swidden fields. In contrast, men and women in the OP site did not see each other for most of the day and worked side-by-side only on Sundays and holidays.

## 5 Discussion

Our study compares men and women's time allocation in villages where livelihoods are based upon oil palm smallholder plasma schemes (OP site) with those in villages That did not adopt oil palm but instead practice relatively traditional swidden agriculture (swidden site). Swidden villages are not directly analogous to OP villages prior to oil palm adoption – livelihoods are likely to have diverged over time – but share common characteristics prior to oil palm expansion in the region. The main livelihoods carried out in swidden villages (forest-based swidden agriculture, rubber agroforestry and NTFP extraction) are the same livelihoods practiced in the oil palm villages prior to the adoption of oil palm (see Supplementary Data: Table A.6). Thus, the comparison in this study between groups represents an exploration of different trajectories from a shared historical baseline.

The core difference between the sites is the greater time spent in off-farm labour in the oil palm site. Both quantitative and qualitative results show substantially more time allocated to off-farm work in the oil palm site compared to the swidden site for both men and women. This increase is predominantly attributable to wage labour on oil palm plantations. In the oil palm site 89.9% of men's and 87.2% of women's time, spent in off-farm labour was spent in oil palm plantation employment. On average, men spent 4.3 h longer in off-farm employment in the oil palm site compared with the swidden site, while women spend 4.5 h longer. Time allocated to oil palm labour necessitates reductions in time elsewhere. Both men and women spent substantially less time in on-farm labour in the oil palm site (with greater reductions for men than women). In addition, women time spent less time in personal and leisure activities as well as sleep. Our qualitive results illustrate how differences in gender roles between sites derive in part from the changing nature of opportunity costs of labour as well gendered consequences of time and labour-saving adaptations employed to mitigate time allocation trade-offs.

### 5.1 Income producing and food producing labour

Livelihoods among smallholder swiddening Dayaks have been described as “*dual or composite economies*” (Dove, 2011) consisting of an income generating set of activities combined with a set of unrelated food producing activities. This characteristic of Dayak smallholder livelihoods has proven remarkably resilient to market forces, cash crop prices and economic opportunities (Höing and Radjawali, 2017). Livelihoods in both sites in this study both resemble this characterisation. In the swidden site, the combination is primarily rubber and swidden (with some NTFP collection), while in the oil palm site the combination is plantation labour and a more sedentary form of rice cultivation. For most households, in both sites, exlcusive focus on income generating activities was not viable, and would not have produced sufficient income to support a family. Likewise, in neither site would subsistence alone be a viable strategy.

While the dual economy strategy exists in both sites, there are essential differences in the way households optimise between income generation and food production. In the swidden site, time and labour are allocated to flexibly, according to need. This is possible because of the nature of income generating activities. Time allocated to rubber tapping or the collection of NTFPs can be easily increased, reduced, paused or restarted. In the OP site however, plantation labour is fixed and inflexible consisting of a minimum number of hours per day at fixed times of the day. The increased time spent in oil palm labour, combined with insufficient income to rely on oil palm alone necessitates time and labour-saving changes to agricultural production. Rubber, which is both time consuming and best collected during early mornings when oil palm labour is carried out, is replaced with pepper. Changes are also made to rice production, as swidden cultivation relocates closer to villages, and fallow lengths are shortened – reducing the need for long walks to and from fields and reducing the labour (primarily men's) required for opening new fields.

Interestingly, we found little evidence that agrarian change resulted from land scarcity due to oil palm expansion. Most respondents still claimed they had access to swidden land and sufficient farmland was available. Likewise, although rubber cultivation had declined significantly, many households still retained rubber gardens. While there may be discrepancies between perceived and legal access and ownership of land due to the erosion of customary land rights (Clerc, 2012), it is clear that changes to agricultural production were motivated, initially at least, not by a lack of land, but by a lack of time. While swidden land was not considered scarce, prime land close to roads and villages was in high demand and local land markets emerged to buy and sell these plots.

### 5.2 Time scarcity, reproductive labour and leisure time

Relative to the swidden site, both men and women in the OP site experienced time scarcity and time pressure. For women, this time pressure resulted in women sacrificing time spent in leisure and rest. Compared with the swidden site, women in the OP site spent less time in personal and leisure activities as well as sleep. The regression results suggest that domestic labour including cooking, cleaning and childcare is relatively inelastic (i.e., it cannot be reduced to compensate for increases in time spent elsewhere). Our qualitative findings indicate that women may sacrifice rest and leisure time to maintain their ability to care for children and carry out other domestic duties. One striking example is women returning home during breaks in plantation labour (often against the instruction of supervisors) to begin cooking mid-day meals. Women in the OP site were also more likely to go to bed after and wake up before their husbands, thus sacrificing sleep and leisure time to prepare breakfast so men could quickly eat before plantation work the next morning.

### 5.3 Gender roles and household gender dynamics

The two sites displayed different patterns in the allocation of time and labour. Compared with the swidden site, women in the OP site took on a greater share of responsibility for household food production. This is reflected both in the relative shares of time spent in own-production for men and women, as well as the wider range of roles carried out in own-production. Likewise, compared with the swidden site, men in the OP site took on a greater share of the responsibility for income production. While in absolute terms the time spent in agricultural production was reduced for men and women, in relative terms, time spent in agricultural and forest-based activities was reduced to a greater extent for men than for women. Thus, it appears that men benefited more than women in terms of reducing agricultural labour and benefited the most from labour-saving efficiencies.

### 5.4 Household-decision making

#### 5.4.1 Participation in off-farm labour

The underlying driver behind changes in time and labour allocation were differences in participation in off-farm labour. Participation in off-farm labour in the swidden site was limited by lack of opportunities. Obtaining regular salaried off-farm work in swidden villages generally requires migrating, at least temporarily. In contrast, in the OP site, both men and women had access to ample opportunities for off-farm work in the form of waged plantation labour, with men also having access to numerous other sources of supplemental off-farm work such as driving trucks and security work.

The degree of participation in plantation labour in the OP site reflects the nature of the plasma scheme model. We explicitly did not investigate contractual arrangements with oil palm companies.^9^ Nevertheless, though classified as plasma smallholder farmers, the livelihoods of respondents more closely resembled that of waged plantation labourers than smallholder farmers. Participants received almost all their income from, and spent almost all their income-generating time in, waged plantation labour for oil palm companies.^10^ Very few plasma participants in our survey farmed their oil palm plot. Our observation that the livelihoods of smallholder plasma scheme participants bear closer resemblance to waged plantation workers than oil palm farmers is matched by numerous other studies among formerly subsistence orientated farmers in Kalimantan (Julia and White, 2012; Bissonnette, 2013; Li, 2015; Elmhirst et al., 2017; Maharani et al., 2019). Such ‘one-roof management’ systems are increasingly common in Kalimantan as companies aim to move away from inefficiencies of out-grower systems and centralise management of plantations (Zen et al., 2016; Hasudungan, 2018), enabled by legal loopholes and a government preference for direct compensation negotiations between affected communities (Gillespie, 2011; Purwanto et al., 2020).

These results highlight the fact that the mechanisms through which oil palm adoption increases participation in off-farm labour may differ by context and smallholder model. Among fully independent smallholders who do not engage in subsistence food production, switching from rubber to oil palm may free up time. In this context therefore, cultivating oil palm as an independent smallholder can be viewed as a “*labour-saving technology*” (Kubitza et al., 2019) which increases men's participation (but not women's) in off-farm labour by freeing up time from agricultural work (Chrisendo et al., 2020). However, in this study, in the context of subsistence farmers whose adoption of oil palm livelihoods is as part of smallholder plasma schemes, participation in off-farm labour is driven not by the labour-efficiency of oil palm, but insufficiency of income generated via plasma dividends.

#### 5.4.2 Opportunity costs of on-farm labour

Different levels of access to off-farm labour creates different opportunity costs of on-farm labour. Results from focus group discussions surrounding household priorities show that households in the OP site aim to maximise income by spending as much time as possible in off-farm labour while also producing sufficient food to meet the bulk of their needs. As oil palm labour was more profitable than competing income generating activities, households aimed to maximise time spent in this activity by reducing time spent in on-farm labour.

The opportunity costs of on-farm labour, however, are different for men than for women. In the OP site men are paid at a higher rate, are offered more hours and have access to a greater variety of off-farm work than women. Similar gendered pay disparities have been found in multiple other accounts of oil palm labour (Julia and White, 2012; Bissonnette, 2013; Li, 2015; Elmhirst et al., 2015, Elmhirst et al., 2017). As a result, households aim to maximise men's time in plantation labour in a joint-utility maximising fashion (Becker, 1965) in order to maximise household income within the constraint of producing sufficient food to feed the family. Labour-saving adaptations to swidden cultivation disproportionately reduce the need for men's time, as well as allowing women to carry out tasks formerly carried out by men. For example, in the swidden site men's roles in transporting heavy goods and cutting and clearing land are time consuming. In the oil palm site, by relocating fields closer to roads and using motorbikes for transportation, the total time required for transportation is reduced as well as allowing women to participate in this previously male-dominated task. Likewise, reducing fallow cycle frequency reduces the need for male labour in land clearing, while the use of herbicides to poison trees instead of cutting allows women to participate in this task. The effect is a shift not only in time allocation but also household gender roles.

These findings support previous qualitative studies that have shown that swidden-oil-palm transitions result in changes in the gendered distribution of labour (Julia and White, 2012; Villamor et al., 2014, Villamor et al., 2015; Li, 2015; Elmhirst et al., 2017; Maharani et al., 2019). For example, we found near identical shifts in gendered labour dynamics to Maharani et al., (2019) who showed how changing gender dynamics were driven by labour-saving adaptations such as changes to the farming system (shortening/skipping fallow periods, relocating fields closer to roads), access to technology (use of chemical inputs, motorbikes) which reduced the need for many forms of physical labour which were previously carried out by men.

#### 5.4.3 Intra-household dynamics and social norms

While decisions to maximise returns on men's labour appear to be made at the household level, we have little data on intra-household decision making. Women's agency in such decisions is a vital component of the process missing in our study (see section 4.6.). Decision making also occurs in context of local social and cultural norms and expectations. Women's social and cultural role in reproductive labour, especially as caregivers and cooks, was seen as immutable and a major factor as to why women could not work longer hours in productive labour. Likewise, greater pay for men than women in oil palm labour was considered uncontroversial by both men and women with respondents typically citing the more physical nature of men's labour. However, men were also given nearly all the positions of less physically demanding, but more prestigous and better renumerated roles such as supervisors and office workers. Women were also seen as lacking the knowledge to mix chemical inputs – despite both men and women applying chemical inputs in plantation labour. It should also be noted, that contractual arrangements are usually the product of village level negotiations between local elites and companies – from which women are often excluded (Julia and White, 2012; Elmhirst et al., 2015; Yuliani et al., 2020). Thus, both levels of pay and access to work are the product of pre-existing cultural and social views on gender roles, filtered through agreements made by village elites on behalf of communities.

### 5.5 Time allocation, swidden transitions and agrarian change

Two main mechanisms drove changes in agricultural production; (1) the replacement of rubber cultivation with less time consuming but more capital-intensive cash crops such as pepper and (2) changes to swidden cultivation such as the relocation away from upland slopes, shortening fallow periods and reducing rotation frequency, and intensification through use of chemical inputs. Agrarian changes such as the progressive abandonment of swidden have wide-ranging consequences for the broader landscape. These changes echo swidden transitions across Southeast Asia (Dressler et al., 2016; Padoch et al., 2007; Fox et al., 2009). Several studies have identified similar transitions in Kapuas Hulu and other regions of Borneo driven first by logging (Colfer, 2008, Padoch et al., 1998, Wadley, 1997) and then by oil palm (Mertz et al., 2013; Maharani et al., 2019). Our results closely match those of Maharani et al. (2019) who found shortening of fallows, relocation of fields closer to villages and increased use of chemical inputs in swidden-oil-palm transitions.

The changing gender dynamics observed in our study are simply the latest development in a long history of changing gender dynamics within swiddening communities in Kapuas Hulu. Changes in gender-dynamics seen during the logging boom share similarities with latter changes in gender-dynamics caused by oil palm's expansion (Elmhirst et al., 2016). Colfer (2008) documented the effects of a nascent logging industry on gender dynamics among swiddening Kenyah Dayaks in East Kalimantan. The parallels with this case study are striking. New off-farm labour opportunities emerged which benefited men more than women leading to men becoming seen as responsible for income generation. At the same time, new technology (such as chainsaws and outboard motors) reduced the workload for men within swidden cultivation but not for women. These changes affected not only the allocation of time and labour but also had lasting effects upon intra-household gender dynamics.

### 5.6 Limitations and future research

A major limitation of this research is the cross-sectional nature of the survey resulting in the potential for endogeneity bias. This can arise if there are unobserved village properties that make it more or less likely to adopt oil palm which also affect the allocation of time and labour in some way. While our best attempts were made to control for this possibility at the site selection stage (section 2.3), there may still be unknown sources of endogeneity and longitudinal studies are necessary to confirm these findings. Longitudinal studies would also be helpful in exploring the effects of seasonality. While we controlled for high-labour periods in the regression model and avoided conducting surveys during burning, planting and harvests, the lack of seasonal data is a major limitation of this study. To explore the effect of seasonality of labour on time allocation, longitudinal studies across seasons, over multiple years are needed. Similarly, future studies should pay close attention to work patterns throughout the week. As formative research indicated that Sundays were a day of rest we did not conduct 24-recall surveys on Mondays. Further investigation, however, reveals that in the OP site, these days off plantation work are used for men and women to work together in own production. Thus, estimates of time allocated to own production are likely to be artificially low in the OP site.

This time use study formed part of a wider investigation into maternal and child diet and nutrition. As a result, the sample was limited to mothers of small children from indigenous Dayak ethnicities and we are unable to explore intersections with numerous other characteristics such as wealth, ethnicity, age, migration and social and political capital. In the OP site, this caveat is particularly pertinent. It has been widely noted that oil palm is a “*rich farmer's crop*” (McCarthy, 2010) – requiring substantial capital investments in seedlings and fertilizers as well as the means to wait between planting and the first harvest. Access to oil palm, therefore often requires prior wealth or access to credit. While in theory smallholder partnership schemes are designed to overcome the technological, knowledge and capital barriers to oil palm adoption among smallholders, participation is still only available for a sub-section of land-owning residents. Wealthier and more politically connected local elites often benefit disproportionately from such schemes (Yuliani et al., 2020). This likely includes access to different types of jobs which can affect the allocation of time. Poorer households are also more likely to sell their plasma stake back to the company or other residents (Li, 2015, Semedi and Bakker, 2014). Our study also overlooks the complex reality of intergeneration dispossession of land (Elmhirst et al., 2017) as well as the reality of migrant labour upon which oil palm production depends (Pye et al., 2012; Elmhirst et al., 2015; Lindquist, 2017; Maharani et al., 2019). Future studies, with larger and more diverse samples of respondents are needed to fully explore these effects alongside the complementary qualitative research needed to interpret these findings.

Inherent in the study design are certain assumptions about how households allocated their time. By modelling shares of time Allocate to different activities, the model highlights individual-level trade-offs in time allocation. The qualitative study identifies household-level time use strategies and decision-making, but does not address the issue of how these decisions are made within the household. Further consideration of intra-household power dynamics and agency over time allocation are needed to contextualise these results. Our study also excludes other members of households such as grandparents, adult and teenage children and other kin relations. The results show that the allocation of some activities (especially childcare) to other household members is an important coping strategy for women faced with time pressure. Consideration of time allocation among other household members as well as wider kin relations is necessary to quantify absolute times spent in different activities. Such surveys would be necessary, for example, to explore whether oil palm affects the total time children spend in childcare.

Mixed methods are highly suited for explorations of changes in labour and time (White, 1984; Stevano et al., 2019). Our study reiterates the importance of mixed methods in studies exploring the effects of oil palm expansion in Indonesia. Mixed method research, though suited to socio-ecological investigations, is underrepresented in oil palm research globally (Reiss-Woolever et al., 2021). In our study, many aspects of the time and labour transition could not be explained by either quantitative or qualitative methods alone. For example, conclusions based just on qualitative data might have under-emphasized the degree to which women's agricultural labour is lower in the OP site in absolute terms. Similarly, conclusions based only on the quantitative data would not have detected the complex set of interrelated decision-making processes nor the physical and mental stress of time pressure experienced by women. Increased use of mixed-method research could mitigate the use of over-simplistic narratives such as the ‘*feminisation of agriculture*’, ‘*liberation from on-farm work*’ or ‘e*ngagement in opportunities for off-farm labour*’ and instead focus on the suite of simultaneous drivers and feedback loops which determine well-being outcomes in contexts of rapid livelihood and landscape change.

Our study also reveals the importance of using robust time use methodologies with full-accounting 24-h recall time use methods able to capture simultaneous activities. Without such methods it is impossible to observe the true effect of oil palm adoption on livelihood changes or the coping strategies employed to cope with time scarcity. Recent research has shown the promise of new and innovative approaches to time use research (e.g., accelerometers and GPS) (Picchioni et al., 2020; Srinivasan et al., 2020). Application of these methods in conjunction With full-accounting time use recall methods could reveal the effects of agrarian and landscape change on energy expenditure and physical exertion. Future research could also benefit from further disaggregation of time use categories – in particular, different types of Waged labour – as well as disaggregation of time spent of agricultural production time by crop type and production system.

## 6 Conclusion

Adopting oil palm based livelihoods creates gendered shifts in the allocation of household time. The effects of smallholder oil palm adoption on intra-household gender dynamics and allocation of time and labour will depend not only on the model of oil palm production but also the baseline conditions and livelihoods of the adopting households. For example, among commercialised farmers in Sumatra, independently switching from rubber to oil palm reduced on-farm labour for both men and women, but increased participation in off-farm labour only for men (Chrisendo et al., 2020). However, for former subsistence farmers in Kalimantan, participating in oil palm plasma schemes increased both men and women’s participation in off-farm labour, though at different rates, leading to shifts in the gendered allocation of household labour (Elmhirst et al., 2015, Elmhirst et al., 2017, Julia and White, 2012, Maharani et al., 2019, Toumbourou and Dressler, 2020)

Building upon previous studies of gendered labour dynamics in oil palm adopting communities, this study uses primary data collected using robust standardised time-research methods among both women and men in oil palm and non-oil-palm communities. The study combines this time use data with qualitative research on men and women's experience of time as well as the causes, experience and management of trade-offs in time allocation between different activities. Our results suggest that oil palm adoption (participation in smallholder plasma schemes) among former swidden farmers drastically alters the intra-household allocation of time and labour. Oil palm adoption is associated with more time spent in off-farm labour for both men and women – but significantly more so for men than for women. Likewise, oil palm adoption is associated with a less time spent in agricultural and forest activities for both men and women – but significantly more so for men than for women. These findings indicate a trade-off between time spent in off-farm labour and time spent in agricultural and forest-based activities. This trade-off is corroborated by the qualitive findings which indicate that households in the oil palm site maximise time spent in off-farm labour and minimise time spent in agricultural and forest-based activities at the household level and shift as much subsistance agricultural labour towards women as possible. This is achieved through a series of changes to agricultural production which interact in complex non-linear ways with broader landscape processes of land use and agrarian change.

The increased time women spend in productive labour in the oil palm comes at the cost of personal and leisure time as well as sleep. Our qualitative findings confirm that women perceived an overall scarcity of time, and that this time pressure manifests itself in the form of mental and physical stress. Time pressure may have significant effects upon maternal and child nutrition as well as subjective well-being and women's empowerment (Kadiyala et al., 2014; Johnston et al., 2015; Stevano et al., 2019). Further research is required to integrate time use with new and emerging measures of subjective well-being (Diener et al., 2018). Investigation of these pathways is urgently needed to fully understand the welfare effects of oil palm adoption among smallholders in Indonesia. At the same time, effects of time pressure on maternal and child nutrition should be explored through the lens of food acquisition and food choice behaviour and through the effect on women's energy expenditure.

Our study indicates that oil palm adoption may have significant implications for gender equity, well-being and maternal and child nutrition via changes in household time allocation. Similar studies using specialised time use methods but using longitudinal study designs are needed to determine whether these findings have general applicability. Our results reflect one specific context, at one specific stage of a broader landscape, agrarian and economic transition. In addition, our sample was restricted to a relatively homogeneous group of ethnically similar, indigenous, land-owning households with mothers of small children. Further investigation of time use effects in different contexts and different models of oil palm adoption are needed, as well as investigations into how wealth, class, age, ethnicity, land ownership, migrant status, and education interact with labour transitions.

## Declaration of Competing Interest

The authors declare that they have no known competing financial interests or personal relationships that could have appeared to influence the work reported in this paper.

## Appendix A Supplementay data

Supplementary material Image 1

## Footnotes

1 For example, independent smallholders are prevalent in many provinces of Sumatra where smallholders oil palm schemes have existed since the 1970s. Independent smallholders in this context may consist of ex-smallholder scheme participants, or commercialised rubber farmers adopting oil palm independently but benefiting from spill-over effects of local oil-palm processing (Euler et al., 2016; Jelsma et al., 2017). In contrast, oil palm expansion in areas with a more recent history of oil palm (concentrated in Papua and Kalimantan) more typically takes the form of corporate estates combined with legally mandidated smallholder partnership schemes (Jong, 2020; EIA, 2021)

2 As discussed in section 2.2.1, definitions of independent smallholders are ambiguous and include a wide range of medium and large scale farmers, absentee farmers and outside investors. Survey data from Central and West Kalimantan suggests that independent oil palm is dominated by local elites and entrepreneurs (both migrant and local) who often lack prior experience of cultivating oil palm, but nevertheless own large plots, farmed often in absentia (Schoneveld et al., 2019a). Additionally, although many independent smallholders own multiple plasma plots, few are themselves former plasma scheme participants but instead acquired plasma plots through purchasing them from plasma scheme participants who abandoned oil palm (Schoneveld et al., 2019b).

3 At the time of writing, changes to this law are being attempted but are still held up in disputes in court. However, even if national law changes, many provincial and district governments have similar requirements.

4 Power relationships are asymmetrical both between companies and communities but also between community elites and other member of the community (Prabowo et al., 2017; Hasudungan and Neilson, 2020). Several studies have shown that local male elites dominate negotiations on behalf of all village residents (Semedi and Bakker, 2014; Elmhirst et al., 2015; de Vos, 2016) with said elites often receiving substantial rewards to persuade others to drop their opposition to oil palm (Yuliani et al., 2020).

5 As FML regression does not work with zeros, in fact one minute was added onto all times to overcome this issue.

6 Collection of rubber is not limited by the area of rubber gardens owned by a household due to a well-established and standardised system of profit sharing whereby households may collect rubber on land owned by others.

7 Rubber yields are not reduced from less frequent harvesting.

8 Both activities require labour in the early mornings. Rubber yields were said to be greatest when temperatures were cooler. Work hours for oil palm labour are set by the company. Plantation labour begins early in the morning.

9 Our study covered too many villages to conduct a detailed investigation of contractual arrangements. Sensitivity around the topic of contractual arrangements with companies was encountered during formative research. To reduce bias in results we therefore explicitly avoided this topic of research. Formative research suggested that contractual arrangements between individuals, communities as a whole and the oil palm company differed between villages. A wide-variety of conflicting opinions and perspectives were heard, but ground truthing claims is extremely difficult without triangulation from multiple sources, alongside long-term ethnographic investigations. Due to the large number of villages, there are likely to be multiple realities for different communities and different sub-populations. Generalisations of this study as a representative of plasma models should not be made.

10 Many respondents received no income whatsoever from plasma farming despite being enrolled in plasma schemes, pointing instead to community development projects paid for by companies and/or housing improvements. Many so-called plasma farmers could not identify the location of their plamsa land, and some were unsure as to whether they owned any. Only 3% of plasma farming households reported revenue from plasma dividends in their top three sources of household income. While we can provide no quantitative estimates of prevelence, a secondary market for plasma land appears to exist and the sale of plasma land either to other farmers or back to the company appears to be far from uncommon.
