# Supplementary material for: Oil palm and gendered time use: A mixed-methods case study from West Kalimantan, Indonesia
Source: For Policy Econ. 2022 Apr;137:102682. doi: 10.1016/j.forpol.2021.102682 (PMC8914600; doi:10.1016/j.forpol.2021.102682)
Supplement: Supplementary file 1 — Supplementary material [file mmc1.docx]

# APPENDIX A: Supplementary Tables

Table A 1: Seasonal swidden calendar in Kapuas Hulu*

|  | January | February | March | April | May | June | July | August | September | October | November | December |
| --- | --- | --- | --- | --- | --- | --- | --- | --- | --- | --- | --- | --- |
| Land Clearing |  |  |  |  |  |  |  |  |  |  |  |  |
| Burning |  |  |  |  |  |  |  |  |  |  |  |  |
| Planting |  |  |  |  |  |  |  |  |  |  |  |  |
| Weeding |  |  |  |  |  |  |  |  |  |  |  |  |
| Harvesting |  |  |  |  |  |  |  |  |  |  |  |  |

* Months shown are ranges during which activities may occur. Activities themselves may take only a period of weeks. Data is based on focus group discussions.

Table A 2: Marginal effects of covariates on time shares in activities.

|  | Wage Work | Agriculture and Forest | Reproductive Labour | Personal and Leisure | Sleep |
| --- | --- | --- | --- | --- | --- |
| Female | -0.067*** | -0.026*** | 0.123*** | -0.026*** | -0.003 |
|  | (0.011) | (0.008) | (0.008) | (0.006) | (0.004) |
| Oil palm | 0.119*** | -0.094*** | 0.007 | -0.015 | -0.016*** |
|  | (0.014) | (0.015) | (0.008) | (0.009) | (0.006) |
| Female employment | 0.135*** | -0.018* | -0.048*** | -0.056*** | -0.013*** |
|  | (0.013) | (0.010) | (0.008) | (0.008) | (0.005) |
| Age | 0.001 | 0.000 | -0.002** | 0.000 | 0.000 |
|  | (0.001) | (0.001) | (0.001) | (0.001) | (0.000) |
| Middle school edu. | -0.013 | 0.008 | 0.005 | 0.006 | -0.005 |
|  | (0.013) | (0.011) | (0.010) | (0.009) | (0.005) |
| High school edu. | -0.004 | 0.003 | 0.001 | -0.003 | 0.003 |
|  | (0.012) | (0.009) | (0.009) | (0.007) | (0.004) |
| High season | -0.001 | 0.004 | 0.010 | -0.023*** | 0.009** |
|  | (0.009) | (0.008) | (0.007) | (0.007) | (0.004) |
| Wealth (asset index) | 0.002 | -0.006** | 0.000 | 0.002 | 0.002* |
|  | (0.003) | (0.003) | (0.002) | (0.002) | (0.001) |
| Children <14yrs | -0.003 | 0.003 | 0.003 | -0.000 | -0.002 |
|  | (0.005) | (0.004) | (0.003) | (0.003) | (0.002) |
| Children 14-18yrs | -0.000 | -0.012*** | 0.008** | 0.002 | 0.002 |
|  | (0.005) | (0.004) | (0.004) | (0.003) | (0.002) |
| Adults >60yrs | 0.006 | 0.000 | 0.004 | -0.014** | 0.004 |
|  | (0.010) | (0.008) | (0.007) | (0.006) | (0.003) |
| Land area (log) | 0.001 | 0.005 | -0.002 | -0.005 | 0.001 |
|  | (0.005) | (0.004) | (0.003) | (0.004) | (0.002) |
| Hired labour | 0.005 | 0.008 | -0.002 | 0.000 | -0.011 |
|  | (0.013) | (0.014) | (0.011) | (0.012) | (0.007) |
| Fertilizer | -0.000 | -0.011 | 0.001 | 0.003 | 0.007 |
|  | (0.015) | (0.018) | (0.013) | (0.012) | (0.008) |
| Pesticide | 0.023 | -0.017 | -0.021** | 0.005 | 0.009 |
|  | (0.014) | (0.014) | (0.010) | (0.010) | (0.006) |
| Herbicide | -0.010 | 0.008 | -0.009 | 0.028*** | -0.017*** |
|  | (0.013) | (0.010) | (0.008) | (0.007) | (0.005) |
| No rice | 0.009 | 0.017 | 0.005 | -0.018 | -0.013* |
|  | (0.013) | (0.017) | (0.011) | (0.012) | (0.008) |
| Grows rubber | -0.030*** | 0.023** | 0.012 | -0.006 | 0.001 |
|  | (0.011) | (0.010) | (0.008) | (0.008) | (0.005) |
| Grows pepper | -0.014 | 0.019 | 0.003 | -0.008 | 0.000 |
|  | (0.013) | (0.019) | (0.011) | (0.010) | (0.007) |
| Chi-squared | 1308.44*** | | | | |
| BIC | 1748.543 | | | | |
| AIC | 1417.883 | | | | |
| N | 603 | | | | |

Note: Fractional Multinomial Logit model. Robust standard errors clustered at household level. *** denotes statistical significance at 1% level, ** at 5% level and * at 10% level.

# Appendix B: Sensitivity Analysis

Table A 3: Sensitivity analysis showing differences in ratios between weighting concurrent primary and secondary activities as 50% each and as 80% primary and 20% secondary.

|  | Weighting 0.5 | | Weighting 0.8 | |  |  |
| --- | --- | --- | --- | --- | --- | --- |
|  | Mean | SD | Mean | SD | t | p |
| Ratio off-farm | 0.19 | 0.15 | 0.19 | 0.15 | -0.09 | 0.92 |
| Ratio forest and agriculture | 0.09 | 0.12 | 0.09 | 0.12 | 0.03 | 0.97 |
| Ratio of reproductive activities | 0.13 | 0.10 | 0.13 | 0.10 | 0.09 | 0.92 |
| Ratio personal and leisure | 0.25 | 0.07 | 0.25 | 0.07 | -0.01 | 0.99 |
| Ratio sleep | 0.34 | 0.04 | 0.34 | 0.04 | 0.03 | 0.97 |

# Appendix C: Selected quotes to illustrate themes

Table A 4: Example quotes for qualitative themes (OP sites)

| **Subjective Experience of Time Allocation** | |
| --- | --- |
| Physical exhaustion, tiredness, energy | *"The rest is not enough, the body aches sometimes." (OP_KI_F_V3)  "By the evening we are already tired. We are already sleepy. We do not have energy [to socialise] and need to wake up in the morning" (OP_KI_F_V12)* |
| Days off | *"Even on Sundays we sometimes go to the fields. There is no rest…”  (OP_KI_F_V9)* |
| Breaks and rest | *"When you come home from work from palm oil, you go home, you rest first, you eat and then take a break. If you have rested already, then we'll go to the fields .It is not possible to go straight to the fields because we are too tired.” (OP_KI_F_V4)  "The [breaks] are not enough, because I come home from work at 2. There is a bit of rest, sometimes, but by 3 o'clockwe mus prepare food, look for clean water to drink." (OP_KI_F_V10)  "We are are busy working but in the afternoon, after coming home from working on palm oil, we have to rest for a while. After then we go to work again" (OP_KI_F_V12)* |
| Busyness / multiple demands | *“You don't have time because ... when we come home from work, we work again to take care of our husbands" (OP_KI_F_V7)  "In the evening working too, working the evening. It is impossible to rest when you are tired. We are pushed for time. If you are resting the work is not continuing. (OP_KI_F_V9)"  "Indeed, there is no time. It is true. We have time, but we use all our all time looking after children. (OP_KI_F_V1)"* |
| Opportunities to socialise | *"How can we have time to get-together?”  (OP_KI_F_V8)  “We hang out in the stall together with the group, so we rest by hanging out. We will spend the time like that and then will return to work. After returning to work, we only rest again at night.” (OP_KI_M_V2)  "...to hang out? It depends, we rarely do that [socialise while working]. Hanging out in groups is rarely done unless there is a wedding or there are [customary events] when we group together to work." (OP_KI_F_V2)* |
| Working as household unit | *"My husband works more [than me] in the oil palm. I go alone to farm land. I go with my children or siblings like that. Usually if we are farming in the garden, we are alone without my husband." (OP_KI_F_V4)* |
| Existence of trade-offs in time allocation (sleep/ rest vs reproductive; productive vs reproductive; paid work vs agricultural work) | "Finding land is not yet a difficulty. For us, the only difficulty is finding time" (OP_KI_F_5)  "We wake up earlier [than husbands], around 4 we wake up, we prepare breakfast and so on for our husbands... so they will be able to directly eat breakfast and immediately go to work" (OP_KI_F_V4)  "Yes sometimes, if we are busy. We will skip the rest" (OP_KI_F_V8)  "Women do lighter work for a shorter time. We go home quicker and usually we divide our time to do other activities such as farming, care for the house, care for children and others." (OP_KI_F_V9)  "The men who work as drivers, they have to work late at night, so men don't have the time to help women unless there is a day off" (OP_KI_F_V6)  "We are too busy. There is no time to grow vegetables or garden vegetables for sale" (OP_KI_F_V7) |
| **Labour and Time Saving Efficiencies** | |
| Swidden changes (relocation of fields, shorter fallows, less frequent rotation, chemical inputs) | *"The first difference is [the fields] are getting smaller, the second is chemicals are used like fertilizer, herbicides, pesticides. It is changing now, before if it was more natural then, now we use of herbicides, pesticides" (OP_KI_M_V4)  "We rarely open [new] land that has large trees. It is too much work. Also, because land is scarce, so we do our farming on the same land" (OP_KI_M_V2)*  *"It [fields] used to be far because we walked. Now it feels close for us because we use a motorbike. Now people think they don't want to have a field far away, because it takes time. Now people think because they are busy working, they will farm closer to their homes so they are easy to maintain, easy to monitor" (OP_KI_FGD_V5)"*  *"Actually, the clearing is easier because the fields have been open for many years." (OP_KI_M_V6)  "It is much better to sawah [permanent unirrigated wet rice]. Like before to give an example, because the fields move around the trees are so big, so we cut down and we burn it down then we clear it, then we will straighten it. For the sawah you do not need to clear. You can do it all with a hoe. Straight after hoeing you plant, it is not like this with moving around." (OP_KI_M_V2)  "Because sawah [permanent unirrigated wet rice] is not too tiring, caring for it is easier and faster. For example, in August we spray, in in September we are planting, the rest is just waiting for the results. If it was a ladang [swidden]. you have to cut down, then clear, then drill, then clean etc., that's the problem." (OP_KI_FGD_V9)  "For fertilizer and chemicals it is really important, yes, because it can speed up work time like that" (OP_KI_M_V6)  "We used to work the fields here manually without using poison [herbicide], without using fertilizer because in the past we did not recognize chemicals and we worked on the fields with a traditional system. But now we are working the in fields after we have used poison [herbicide] or fertilizer so the process time is faster (OP_KI_M_8)  "For example, if we do it manually, traditionally, it takes one month. But now we use herbicides, with that it is much faster, for example, 2 weeks becomes two days" (OP_KI_M_V9)* |
| Outsourcing of childcare | *"If we are working in the oil palm, [the children] come home from school and go to their grandmother's place, sometimes they are alone." (OP_KI_FGD_V9)  “When the child is left at the company place, usually women will bring provisions for the children. If women do not have time to bring provisions for their children, usually there are people who sell food so that women can buy the food and leave it with the security guards and the guards can then provide food to their children.” (OP_KI_FGD_V2)   “There are people who take care of our children when we go to oil palm, if like us there is no one to look after children at home” (OP_KI_F_V8)  " So it usually depends if they [women] work in the company and there are people at home to look after them. Usually they prefer to leave their children with family at home, but for example they want to work in a palm oil company, they have children and no one takes care of them at home they can bring them to day-care at the palm company" (OP_KI_F_V1)  " ..this day-care was made a long time ago by palm oil companies and there are people who can look after children when left by working women. The [day-care staff] there have been paid by palm oil companies like that" (OP_KI_F_V12)* |
| Use of chemical inputs |  |
| **Gender Roles in Agriculture** | |
| Technical knowledge of machinery and chemicals | *" For women, they cannot operate the machine, because the women here do not care enough to learn, because when dealing with machines they think it is too heavy" (OP_KI_F_V7)  "Normally the men do the clearing using a machine, because it is a machine it is more normally the men." (OP_KI_FGD_V2)* |
| Physical labour | *"The work done by women is a little light work, a little easy. For the work done by the men, the work is more difficult. For example, women, weeding, planting, harvesting, like that. When they are clearing the land, the women weed the grass, the men cut down the trees – the work is more difficult ... When it is harvest, the women can harvest but the men help harvest and transport the harvest from the fields to the house, it's a hard job to transport all the crops to the house. Then the men use the machine to thresh rice and then the men transport the rice that [been milled]. The women take care of the rice which was dried in the sun." (OP_KI_F_V9)* |
| **Time Use Coping Strategies** | |
| Supervision of children while working | *“When we were children, we were more free before. I saw it, children could play in the surrounding natural area and their parents did not forbid it because they can freely play. It is different from the children who are here because the company is polluting and so on, so the children play more at home or children are left with someone who can look over them. The environment is not safe like that.” (OP_KI_F_V3)* |
| Select foods quicker to cook | “*If there is a busy day, maybe we cook eggs or fried noodles. It's the most practical and the easiest to get. If it's hard to buy noodles, just eggs. We can cook and eat that right away.” (OP_KI_F_V5)* |
| Use quicker cooking methods | “*Before we used to cook using firewood and kerosene stoves, now we use gas canisters to make it faster to cook. To get the gas stove, there is help from the government and buy it in palm oil.”* (OP_KI_F_V5) |
|  |  |
|  |  |
| **Household Decision Making** | |
| Household decision making | *“Both [men and women] try to earn money. Only it is more for the men, the men have to earn money, but she only helps. If she can get money, it is okay. But if she can't get money then she will think - he must go earn money” (OP_KI_F_V3)* |
| Seasonal demands of rice production | *“Because of fear of rain, the rice will get wet. So usually wives will coordinate with husbands when it is the harvest season. We will coordinate, either the husband takes time off work and he will help the wife to harvest the land, or the second option, the father continues to work in oil palm but the father provides money and which is given to the mother to pay more people so that the harvest is finished quickly” (OP_KI_F_V12)* |
| Gendered access to labour, contractual terms and pay | *"There is supervisior work, he is under the foreman, a clerk is in charge of attending people who work like that. It is like that, these are the good jobs, but only for men. Men can do these jobs. Women are not able." (OP_KI_FGD_V9)  "Men get paid more. Men can do heavy and non-heavy work, they work longer hours and work faster." (OP_KI_F_V4)  "Nothing [paid leave], so we report that we will take a one-week leave, we set a date from when to when, so as long as they we are on leave, we do not get a salary from the company" (OP_KI_M_3)* |
| Prioritising of men's labour (based upon contractual / pay differences) | *"My husband leaves early in the morning and comes back home at night, or late afternoon. Sometimes when he works as a driver he has to work late at night. So he doesn't have time to work with me unless there is a day off. It is like this, when men are busy with their work activities, automatically the women do the farming." (OP_KI_F_V6)  "The men work for a long time, men have higher salaries because men's work is also heavier than women's. Women's income is smaller than men because women share time, for example, when they are in the fields. When they work on the farm usually they will take time off and not work in a palm oil company, so that the salary obtained by women are smaller than the men." (OP_KI_F_V10)  "The men are more senior because they work in one but and if the women works in a job it is for a short time so she can go home and can work in other activities such as farming and other activities like that." (OP_KI_FGD_2)* |
| Prioritising men's off-farm labour based upon gender roles (men as income producers / women as caregivers) | *Men look for and many other jobs besides farming because they have to earn money, they have to meet the needs of school children and so on, so men are more willing to divide their time to make money (OP_KI_F_10)  "Men can work in all kinds of jobs for the oil palm company because men are more able and men only work in companies. They do not need to do other work, such as taking care of household activities, farming, etc." (OP_KI_F_V7)* |
| Gendered consequences of swidden changes | *"The other reason is because of time. For example, in weeding work, weeding takes longer. In 1 hectare there is more grass than trees. If there are only a few trees, maybe weeding 1 hectare takes a woman 1 week but the man only 2 days.... For burning land in 1 day it is finished, so the men work for a short amount of time and women can work long hours in the fields."* |
| **Consequences of Time Allocation** | |
| Loss of Soil Fertility | *“In the past it was not necessary to use fertilizers, because of fertile soils, if now the soil is less fertile, we need fertilizer” (OP_KI_M_12)*  *“Like my brother-in-law. First, when he opened the land he didn't need spray, no fertilizer, they cut it, after they cut it they hoe it, and it is ready to plant. But after the harvest they need to give compost and fertilizer to plant the soil again” (OP_KI_M_8)* |

# Appendix D: Codebook for Thematic Analysis

Table A 5: Codebook for thematic analysis

| No. | **Theme/ Sub-Theme** | **Description** |
| --- | --- | --- |
| **Subjective Experience of Time** *Experience of time use, effect of time allocation decisions, physical and mental and wellbeing effects of time allocation* | | |
| 1 | **Breaks and Rests** | **Breaks and rest periods during and between work** |
| 2 | **Daily and Weekly Routines** | **Daily routines for men and women. Patterns of time allocation throughout the week.** |
| 3 | **Excess Time** | **Activities carried out when there is extra time. Indicators of excess time. Experience of having sufficient time.** |
| 4 | **Intersectionality** | **Intersections of gender with wealth, class, education, age, ethnicity etc.** |
| 5 | **Seasonal Fluctuations in Time Allocation** | **Seasonal changes in time and labour allocation. Peak and low periods of labour-demand. Swidden cycles.** |
| 6 | **Socialising** | **Opportunities to socialise with others during work or leisure time** |
| 7 | **Time pressure OR Time Scarcity** | **Lack of time, business, stress over lack of time** |
| 8 | **Variety and drudgery of work** | **Perceived variety of work, satisfaction or dissatisfaction with variety of work** |
| 9 | **Weekends and days off** | **Availability and use of weekends and days off** |
| **Trade-offs in Time Allocation** *Perceived trade-offs, conflicts, clashes, incompatibility of time spent, between time allocated in different activities* | | |
| 10 | **Childcare and Productive Labour** | **Trade-offs between child care activities and productive labour (paid work or own-production activities)** |
| 11 | **Domestic Work and Productive Labour** | **Trade-offs between domestic work (reproductive labour excluding childcare) and on and off-farm labour** |
| 12 | **Income Generation and Food Production** | **Trade-offs between time spent in income generating activities and food producing activities** |
| **Managing Trade-offs** Strategies, efficiencies, coping strategies and changes to livelihoods to manage/ mitigate trade-offs in time allocation | | |
| 12 | **Coping Strategies** | **Coping strategies employed to reduce time pressure / manage busy periods / increase efficiency in time allocation** |
| a | Activity Bundling | Combining multiple activities to increase efficiency / reduce time. Concurrent childcare, concurrent food acquisition etc. |
| b | Substitution of activities | Substitution of activities with quicker versions. E.g. reducing cooking time through selection of quicker coking foods / use of quicker cooking fuels |
| 13 | **Efficiencies Adaptations or Changes to cash crop production** | **Substitution of crops / changes in cultivation practices** |
| 14 | **Efficiencies Adaptations or Changes to food production** | **Changes to rice production specifically cited as resulting from time-allocation trade-offs** |
| a | Extra Household Labour | Labour from outside household. Reciprocal Labour exchange or hired labour. |
| b | Modifications to swidden systems | Modifications made to traditional swidden systems to increase time/labour efficiency. Includes changes in fallow length, rotation frequency, relocation of fields closer to villages and roads |
| c | Use of Chemical Inputs and machinery | Use of fertiliser, pesticides and herbicides to reduce time and labour / increase efficiency. Use of machinery and motorised transportation. |
| **Household Decision Making** Factors affecting household decision making in making time allocation decisisions | | |
| 13 | **Opportunity Costs of Agricultural Labour** | **Factors affecting opportunity costs of on-farm labour. Differential opportunity costs for men and women.** |
| a | Access to off-farm labour | Access to off-farm labour opportunities. Access to overtime work. Access to higher-paying office jobs and supervisory positions. |
| b | Contractual Arrangements | Contractual arrangements such as pay, job security, flexibility of work (ability to start-stop-work), contracted hours. conditions, bonus systems etc. |
| c | Gendered Wage Gap | Pay differential between men and women |
| d | Other sources of off-farm income (non-employment) | Off-farm income from non-employment activities. Sale of NTFPs, handicrafts, small business activities etc. |
| 14 | **Household Level Strategies** | **Household level strategy / livelihood approach** |
| a | Flexible allocation of labour | Flexible allocation of labour between income and food producing activities. Desire / ability / practice of switching between income producing activities and food producing activities in response to perceived current / future household demands. |
| b | Gendered Division of Labour | Household decision making to allocate time and labour along gendered lines |
| c | Risk Tolerance and Risk Mitigation | Tolerance of risk, desire for diversified income and food sources, ability to cope with shocks, poor harvests etc. |
| 15 | **Perceived Capabilities and Competencies** | **Gendered perspectives on capabilities / competencies in different types of work** |
| a | Physical Demands of Labour | Activities requiring physical strength and/or strategies to overcome strength requirements |
| b | Specialised techical knowledge | Specialised technical knowledge required for operation of machinery / mixing chemicals |
| c | Women's Reproductive Roles | Women’s assumed dominance and superior competence in reproductive labour |
| **Effects of Managing Trade-offs in Time Allocation** Consequences attributed to changes in livelihoods which occurred as a result of managing trade-offs in time allocation | | |
| 16 | **Gender Roles** | **Changes in gender roles and responsibilities stemming from modifications made to manage time allocation trade-offs** |
| 17 | **Land Markets and Land Scarcity** | **Emergence of land market in location of relocated agriculture** |

# Appendix E

# **Study site selection and potential sources of endogeneity**

Comparison of oil-palm and non-oil palm adopting villages

We opted for comparisons between randomly selected households within oil-palm and non-oil palm villages, as opposed to the random selection of oil-palm and non-oil palm adopting households within villages with both oil-palm and non-oil palm households for the following reasons: (1) Comparisons of oil-palm and non-oil palm adopting households within mixed villages are likely to suffer from important omitted variable biases due to difficulty for controlling for such important potential issues such as a household’s political connectedness and local influence in land use and land rights decisions; (2) comparisons between oil-palm and non-oil palm adopting households are likely to suffer from survivorship bias with households who were successful at oil palm being over-sampled while unsuccessful oil palm farmers risk being categorised as non-oil palm adopters. This effect likely increases with time as unsuccessful farmers sell their land to more successful farmers and adopt alternative livelihoods or migrate out of villages. (3) Oil-palm plasma agreements are made at community levels – with consent being granted by village authorities on behalf of village residents and where dividends, compensation and other forms of payments are collectively bargained; (4) non-oil palm livelihoods are dependent on diverse landscape mosaics of forests, fallows, agroforests and fields which may be reduced or lost after oil palm development; (5) oil palm often precipitates a loss of customary rights and land tenure upon which forest-based swidden livelihoods depend.

Potential Sources of Endogeneity

The decision or opportunity (or lack thereof) to adopt oil palm is not random. There are five potential reasons why oil palm may not have been developed in a particular village; (1) a community may have been approached by an oil-palm company but rejected the company offer and terms; (2) the area is unsuited to oil palm cultivation due to poor soils or steep slopes; (3) it is not commercially viable to grow oil palm in the area due to poor infrastructure and market access; (4) government permits cannot be obtained to grow oil-palm in the area due to land use zoning as conservation forest, national park, or as being allocated to other-non oil-palm land uses (e.g. forestry); (5) a village is situated too far away from existing oil palm plantations and mills. Each of these factors introduces potential sources of endogeneity if differences between oil palm and swidden villages in these characteristics also affect the allocation of labour and time.

We identified five categories of potential endogeneity which were used to create study site selection criteria: cultural, geographical, economic and political endogeneity. These potential sources of endogeneity are discussed briefly below and steps taken to a mitigate them are shown in Table A7.

**Cultural endogeneity** would exist if communities in villages that adopted oil palm differed from communities’ villages which did not adopt oil palm in terms of their ethnic make-up or social and cultural laws and traditions which affected both the likelihood of a community rejecting oil-palm companies as well as the range of agricultural and other livelihood activities carried out. One example of cultural endogeneity might be differences at the baseline period in customary rules and practices. Stronger customary laws over land tenure could lead to a rejection of oil palm companies whilst also affecting the likelihood that certain livelihoods were practiced such as NTFP extraction or rotational shifting cultivation. Differences in these livelihood activities would then explain differences in time and labour allocation.

**Geographical endogeneity** would exist if oil palm and non-oil palm adopting villages differed at the baseline period in such a way that affected the viability of oil palm development or the likelihood of the issuance of government permits to grow oil palm as well as the allocation of time and labour at the baseline period. Examples of geographical endogeneity include current land-use practices and industry (e.g., the presence of logging) which may have affected livelihood occupations or if different slopes or soil types prevented certain types of agricultural production.

**Economic endogeneity** would exist if, at the baseline period, oil-palm and non-oil palm adopting villages differed in terms of wealth, access to financial services such as credit, infrastructure and market access I such a way that it also affected the allocation of time and labour. For example, communities with better access to markers and better access to financial services might be more likely to engage in cash crop production which affected the allocation of time and labour.

**Political endogeneity** would exist if, at the baseline period land use zoning by government authorities which affected whether oil palm could be developed in an area in or around a village also affects the livelihood options available to any particular community. For example, if land was classified as a national park on conservation area which prevented both oil palm development but also slash and burn agriculture.

Village Characteristics Prior to Oil Palm

Table A6 shows a comparison of village level data between the two sites in 1996, the earliest available date of publicly available village data. In every village included in the study, the main occupation of the majority of respondents was reported as food-producing agriculture in 1996. No villages in the study had irrigated rice in the year 1996, and villages in each site had similar areas of non-irrigated rice per household, and similar areas of plantation (including rubber agroforestry) per household. There was a higher (though not statistically significant) area of non-rice agricultural fields in the OP site compared with the swidden site – but this category is broad, encompassing a wide range of land uses. There were no major differences between oil palm and swidden villages in terms of infrastructure and market access. Both sites had a mixture of villages which were primarily accessible by boat and villages which were accessible by road. In the case of villages accessible by road, all were roads from soil or other materials with no villages having stone or asphalt roads. The time to a permanent market via usual transportation was similar in most villages except for two villages in the OP site which were slightly further away.

| \|  \| Forest (Mean) \| (SD) \| OP (Mean) \| (SD) \| \| --- \| --- \| --- \| --- \| --- \| \| Demographics \|  \|  \|  \|  \| \| Number of Households \| 363.17 \| 84.24 \| 347.5 \| 175.66 \| \|  \|  \|  \|  \|  \| \|  \|  \|  \|  \|  \| \| Livelihoods \|  \|  \|  \|  \| \| Main occupation agriculture (% of villages) \| 100 \| - \| 100 \| - \| \| Proportion of Households Farmers (main occupation) \| 0.9 \| 0.09 \| 0.94 \| 0.03 \| \| Main agricultural sub-sector = Food Crops \| 100 \| - \| 100 \| - \| \|  \|  \|  \|  \|  \| \| Agriculture and Land Use \|  \|  \|  \|  \| \| Total Village Area (ha) \| 79911.5 \| 118196 \| 93511.25 \| 94561 \| \| *Village Locality* \|  \|  \|  \|  \| \| -Hill Area \| 50 \| - \| 50 \| - \| \| - *Non-Hill Area* \| 50 \| - \| 50 \| - \| \|  \|  \|  \|  \|  \| \| *Area of land (ha per hh):* \|  \|  \|  \|  \| \| - Rice (any) \| 1.03 \| 0.26 \| 0.99 \| 0.44 \| \| - Irrigated Rice \| 0 \| - \| 0 \| - \| \| - Unirrigated Rice \| 2.06 \| 0.51 \| 2.34 \| 0.99 \| \| - Non-rice Agriculture \| 8.29 \| 1.47 \| 15.67 \| 10.59 \| \| - Plantation (including rubber agroforestry) \| 93.32 \| 132.38 \| 90.22 \| 74.51 \| \|  \|  \|  \|  \|  \| \| Infrastructure & Market Access \|  \|  \|  \|  \| \| *Village Access (% of villages):* \|  \|  \|  \|  \| \| -Main access via road \| 0.67 \| - \| 0.5 \| - \| \| -Main Access via Boat \| 0.33 \| - \| 0.5 \| - \| \| *Type of road (if present)* \|  \|  \|  \|  \| \| - Soil/earth \| 100 \| - \| 100 \| - \| \|  \|  \|  \|  \|  \| \| *Market Access:* \|  \|  \|  \|  \| \| Time to nearest market^2^ (hours) \| 44.8 \| 16.59 \| 66.29 \| 36.04 \| \| *Perceived access to market:* \|  \|  \|  \|  \| \| - difficult/very difficult^3^ \| 100 \| - \| 100 \| - \| \| Market in villages (% of villages) \| 0 \| - \| 0 \| - \| \|  \|  \|  \|  \|  \| \| Financial Services \|  \|  \|  \|  \| \| A*ccess to credit:* \|  \|  \|  \|  \| \| Bank Services \| 0 \| - \| 0 \| - \| \| Credit Unions \| 0 \| - \| 0 \| - \| |
| --- | --- | --- | --- | --- | --- | --- | --- | --- | --- | --- | --- | --- | --- | --- | --- | --- | --- | --- | --- | --- | --- | --- | --- | --- | --- | --- | --- | --- | --- | --- | --- | --- | --- | --- | --- | --- | --- | --- | --- | --- | --- | --- | --- | --- | --- | --- | --- | --- | --- | --- | --- | --- | --- | --- | --- | --- | --- | --- | --- | --- | --- | --- | --- | --- | --- | --- | --- | --- | --- | --- | --- | --- | --- | --- | --- | --- | --- | --- | --- | --- | --- | --- | --- | --- | --- | --- | --- | --- | --- | --- | --- | --- | --- | --- | --- | --- | --- | --- | --- | --- | --- | --- | --- | --- | --- | --- | --- | --- | --- | --- | --- | --- | --- | --- | --- | --- | --- | --- | --- | --- | --- | --- | --- | --- | --- | --- | --- | --- | --- | --- | --- | --- | --- | --- | --- | --- | --- | --- | --- | --- | --- | --- | --- | --- | --- | --- | --- | --- | --- | --- | --- | --- | --- | --- | --- | --- | --- | --- | --- | --- | --- | --- | --- | --- | --- | --- | --- | --- | --- | --- | --- | --- | --- | --- | --- | --- | --- | --- | --- | --- | --- | --- | --- | --- | --- | --- | --- | --- | --- | --- | --- | --- | --- | --- | --- | --- | --- | --- | --- | --- |
| Table A 6: Descriptive data showing differences between oil palm adopting and non-oil palm adopting villages at a historical baseline prior to oil palm adoption. Based upon village level data from PODES 1996 (BPS, 1996). (1) Main occupation of the majority of village residents (2) Time to market with permanent building (3) Subjective rating of easyness / difficulty of accessing market with permanent building (likert scale). |

| \| **Class of Endogeneity** \| **Reason for adopting / not adopting oil palm** \| **Potential Effect Upon Allocation of Time and Labour** \| **Selection Criteria** \| \| --- \| --- \| --- \| --- \| \| **Cultural** \| Rejection of oil palm stronger in some cultural groups or in communities with stronger influence of customary rights and laws \| Different ethnic and cultural groups may specialise in different livelihoods \| - All villages to be majority ethnic Dayaks at both baseline and survey period - No transmigrant villages or villages with significant immigration included - Similar cultural traditions regarding customary practices surrounding agriculture, forest use and livelihoods \| \| **Geographical** \| Oil palm not viable due to steep slopes / poor soils \| Different crops / livelihood activities are viable/ not viable \| - Similar gradients and soil types in all villages \| \| Different forest cover levels at baseline \| Different livelihood options available \| - All villages heavily forested at baseline period. No history of logging or large-scale plantation agriculture. \| \| **Economic** \| Current livelihood practices affects probability of community giving or refusing consent to oil palm companies \| Differences in livelihoods affect allocation of time and labour \| - All villages at baseline period predominantly engaged in swidden agriculture combined with NTFP extraction and smallholder rubber agroforestry \| \| Wealthier villages more likely to accept/reject oil palm \| Households will allocate time differently in wealthy villages from less wealthy villages due to livelihood opportunities \| - Similar levels of village wealth at baseline period \| \| Villages with poor market access less likely to be desired as OP sites \| Differences in market access may affect opportunities for commercialised agriculture and livelihood opportunities \| - All villages similar levels of market access in terms of both time and difficulty \| \| Different access to financial and credit services affect alternative non-oil palm livelihood opportunities \| Differences in access top credit may affect opportunities for commercialised agriculture and livelihood opportunities \| - All villages have similar levels of access to financial and credit services \| |
| --- | --- | --- | --- | --- | --- | --- | --- | --- | --- | --- | --- | --- | --- | --- | --- | --- | --- | --- | --- | --- | --- | --- | --- | --- | --- | --- | --- | --- |
| Table A 7: Potential sources of endogeneity and selection criteria |
